# Robustness of radiomics within photon-counting detector CT: impact of acquisition and reconstruction factors

**DOI:** 10.1007/s00330-025-11374-x

**Published:** 2025-01-31

**Authors:** Huan Zhang, Tingwei Lu, Lingyun Wang, Yue Xing, Yangfan Hu, Zhihan Xu, Junjie Lu, Jiarui Yang, Jingshen Chu, Benyan Zhang, Jingyu Zhong

**Affiliations:** 1 Department of Radiology, Ruijin Hospital, Shanghai Jiao Tong University School of Medicine, Shanghai, China; 2 Department of Pathology, Ruijin Hospital, Shanghai Jiao Tong University School of Medicine, Shanghai, China; 3 Department of Imaging, Tongren Hospital, Shanghai Jiao Tong University School of Medicine, Shanghai, China; 4 Siemens Healthineers, Shanghai, China; 5 Department of Epidemiology and Population Health, Stanford University School of Medicine, Stanford, CA USA; 6 Department of Biomedical Engineering, Boston University, Boston, MA USA; 7 Department of Science and Technology Development, Ruijin Hospital, Shanghai Jiao Tong University School of Medicine, Shanghai, China

**Keywords:** Radiomics, Multidetector computed tomography, Reproducibility of results, Phantoms (imaging)

## Abstract

**Objectives:**

To assess the impact of acquisition and reconstruction factors on the robustness of radiomics within photon-counting detector CT (PCD-CT).

**Methods:**

A phantom with twenty-eight texture materials was scanned with different acquisition and reconstruction factors including reposition, scan mode (standard vs high-pitch), tube voltage (120 kVp vs 140 kVp), slice thickness (1.0 mm vs 0.4 mm), radiation dose level (0.5 mGy, 1.0 mGy, 3.0 mGy, 5.0 mGy, vs 10.0 mGy), quantum iterative reconstruction level (0/4, 2/4, vs 4/4), and reconstruction kernel (Qr40, Qr44, vs Qr48). Thirteen sets of virtual monochromatic images at 70-keV were reconstructed. The regions of interest were drawn with rigid registrations. Ninety-three radiomics features were extracted from each material. The reproducibility of radiomics features was evaluated using the intraclass correlation coefficient (ICC) and concordance correlation coefficient (CCC). The variability of radiomics features was assessed by coefficient of variation (CV) and quartile coefficient of dispersion (QCD).

**Results:**

The percentage of features with ICC > 0.90 and CCC > 0.90 were high when repositioned (88.2% and 88.2%) and tube voltage was changed (87.1% and 87.1%), but none of the features with ICC > 0.90 and CCC > 0.90 when high-pitch scan and different slice thickness were used. The percentage of features with CV < 10% and QCD < 10% were high when repositioned (47.3% and 68.8%) and tube voltage was changed (64.2% and 71.0%), but that with CV < 10% and QCD < 10% were low between standard and high-pitch scans (16.1% and 26.9%) and slice thickness (19.4% and 29.0%).

**Conclusions:**

The PCD-CT radiomics was robust to tube voltage, radiation dose, reconstruction strength level, and kernel, but brittle to high-pitch scan and slice thickness.

**Key Points:**

***Question*** *The stability of radiomics features against acquisition and reconstruction factors within PCD-CT should be fully determined before academic research and clinical application*.

***Findings*** *The radiomics features are robust against tube voltage, radiation dose, reconstruction strength level, and kernel within PCD-CT but brittle to high-pitch scan and slice thickness*.

***Clinical relevance*** *The high-pitch scan and slice thickness that influence voxel size should be set with careful attention within PCD-CT, to allow a higher robustness of radiomics features before the implementation of radiomics analysis in clinical routine*.

## Introduction

Radiomics, a series of analysis methods that translate images into data, has been introduced into radiology more than a decade ago [1–4]. The number of radiomics papers is increasing and the authors of radiomics papers are becoming more diverse in specialties, indicating that the interest in the radiomics technique is continuously growing [5–7]. It has shown great clinical potential for multiple purposes, including diagnosis [8, 9], stratification [10, 11], staging [12, 13], prognosis [14, 15], and treatment response [16, 17]. To standardize radiomics research, our community has drafted and published guidance for transparent reporting and methodological quality for radiomics research, emphasizing the clinical translation of the radiomics models from academic research methods into clinically applicable tools [18–21]. However, radiomics models with generalizability and transferability are still lacking [22, 23]. To bridge the gap between research papers and clinical practice, one important premise is that the robustness of radiomics features should be guaranteed [24–27].

The robustness of radiomics features has been investigated in different imaging modalities [28–32]. The CT radiomics robustness started with the study concerning the acquisition and reconstruction parameters within conventional CT systems, showing the influence of these parameters on the radiomics feature values [33, 34]. Later studies evaluated the consistency among dual-energy CT systems, indicating the great variation of radiomics features due to different dual-energy CT techniques and parameters [35–39]. The reproductivity of radiomics features between and within conventional CT and dual-energy CT systems are also assessed, suggesting that their images are hardly inter-changeable as there are significant differences in radiomics feature values [40, 41]. Unlike the energy-integrating detector CT (EID-CT), the photon-counting detector CT (PCD-CT) directly transfers the X-ray photon into electronic signals without the intermediate step of visible light [42–44]. As a recently available technology, only a small number of literature on PCD-CT radiomics is available [45–49], and the corresponding radiomics robustness studies in PCD-CT are limited. Research on the robustness of radiomics features between PCD-CT and EID-CT has been conducted. Not surprisingly, the radiomics features have a limited level of stability among PCD-CT and dual-energy CT systems [50–53]. Further, the test-retest stability [54], and influence of spectral reconstruction settings [55–57] have been demonstrated within PCD-CT. However, the change in radiomics feature values due to acquisition and reconstruction parameters within the PCD-CT system has not been fully explored.

Therefore, the current study aimed to assess the impact of acquisition and reconstruction factors on the robustness of radiomics within the PCD-CT system.

## Materials and methods

### Study design and phantom

**Fig. 1:**
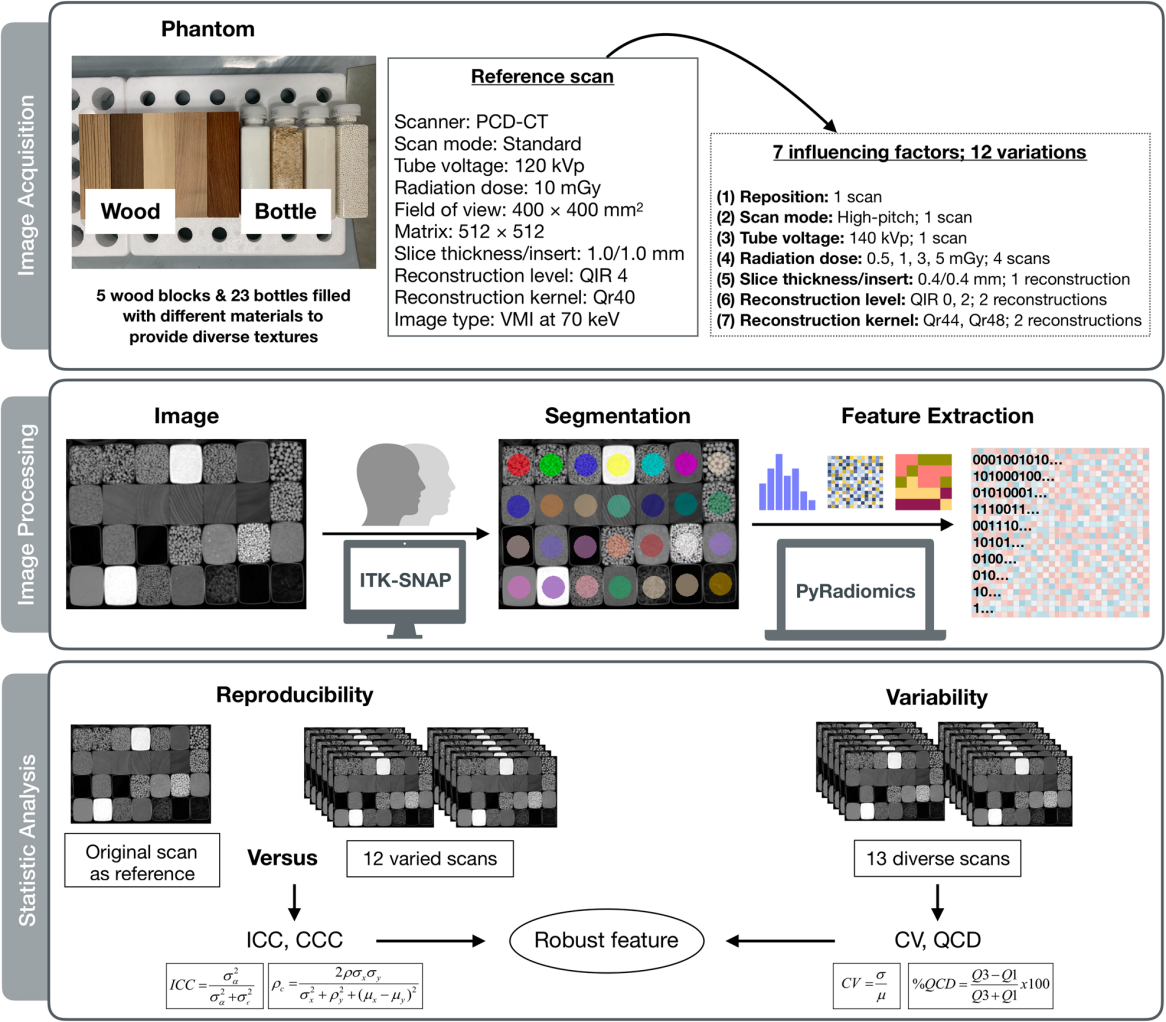
Study workflow. This study consisted of three steps: image acquisition, image processing, and statistical analysis. A home-made texture phantom was scanned with diverse acquisition and reconstruction parameters. The raw data of all scans were generated into VMIs at 70 keV. We extracted 18 first-order and 75 texture radiomics features via Pyradiomics using ROIs segmented with a rigid registration. The repeatability and reproducibility were evaluated by ICC and CCC. The variability was estimated by CV and QCD

**Fig. 2:**
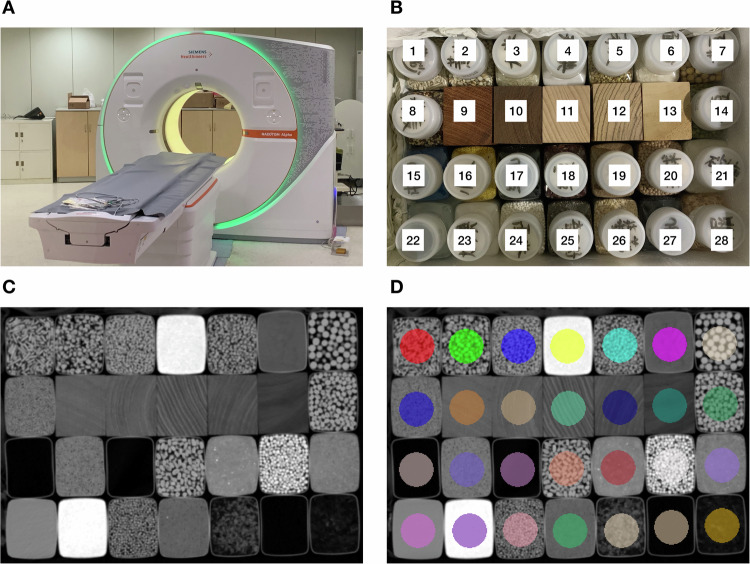
Phantom scan and image segmentation. **A** The homemade phantom was scanned on a clinically available PCD-CT system. **B** The inserts of the homemade phantom were made of wood blocks and bottles filled with different materials. The inserts were placed in a foam plastic box, and kept stable across all scans. The inserts used in current study were: (1) cat litter, (2) coix seed, (3) rice, (4) iodize free salt, (5) buckwheat, (6) flour, (7) soybean, (8) quinoa, (9) rosewood, (10) chicken wing wood, (11) beechwood, (12) zebra wood, (13) basswood, (14) mung bean, (15) mesoporous sponge, (16) millet, (17) microporous sponge, (18) red bean, (19) sand, (20) nutritive soil for succulent plants, (21) granulated sugar, (22) water, (23) iodized salt, (24) sago, (25) chia seed, (26) oat, (27) coarse-pore sponge, and (28) sawdust. **C** CT image of a representative axial slice of the phantom at window level of 0 Hu and window width of 2000 HU. **D** A total of twenty-eight regions of interest were manually contoured on the reference scan, and then copied to all other scans with a rigid registration

The workflow of this phantom study is summarized (Fig. 1). The ethics approval and written informed consent are not required, as the current study was a phantom study. A phantom is designed to provide different textures for analysis (Fig. 2) [33, 51]. The phantom consists of 28 types of materials including five wood blocks and 23 bottles filled with different materials. The inserts in the phantoms are the same as those used in our previous study [51], but the position of these inserts was slightly different from the previous phantom. The materials were positioned to avoid beam-hardening artifacts, and were kept unchanged among all the scans. The detailed setups of the phantom are described in Supplementary Note S1.

### Image acquisition and reconstruction

The phantom was scanned on a PCD-CT system (NAEOTOM Alpha, Syngo. CT version VA50-SP1, Siemens Healthineers) using a reference protocol and protocols with changing acquisition and reconstruction factors (Table 1). The reference protocol was designed to present a typical abdomen-pelvic examination. After the reference scan, the phantom was scanned with changes of acquisition and reconstruction factors in protocols one factor per scan as follows: reposition, scan mode (standard vs high-pitch scan), tube voltage (120 kVp vs 140 kVp), slice thickness (1.0 mm vs 0.4 mm), radiation dose level (0.5 mGy, 1.0 mGy, 3.0 mGy, 5.0 mGy, vs 10.0 mGy), quantum iterative reconstruction (QIR) level (0/4, 2/4, vs 4/4), and reconstruction kernel (Qr40, Qr44, vs Qr48). All the raw data were reconstructed into virtual monochromatic images (VMI) at 70 keV using a vendor-specific workstation (Syngo.Via version VB60, Siemens Healthineers). The energy level of 70 keV was selected as it was the clinical standard of reference at our institution and was considered to be comparable to conventional CT images [58–60].

**Table 1:** Acquisition and reconstruction factors

Influencing factor	Scanner	Scan mode	Tube voltage, kVp	Milliamperage, mAs	Slice thickness/increment, mm	Matrix	Field of view, mm	Rotation time, s	Pitch	Volume CT dose index, mGy	Iteration algorithm	Reconstruction kernel
Reference	PCD-CT	Standard	120	127	1.0/1.0	512	400	0.5	0.8	10.00	QIR 4/4	Qr40
Reposition	PCD-CT	Standard	120	127	1.0/1.0	512	400	0.5	0.8	10.00	QIR 4/4	Qr40
High-pitch scan	PCD-CT	High-pitch	120	127	1.0/1.0	512	350	0.25	3.2	10.00	QIR 4/4	Qr40
Tube voltage	PCD-CT	Standard	140	87	1.0/1.0	512	400	0.5	0.8	10.00	QIR 4/4	Qr40
Slice thickness	PCD-CT	Standard	120	127	0.4/0.4	512	400	0.5	0.8	10.00	QIR 4/4	Qr40
Dose 0.5 mGy	PCD-CT	Standard	120	6	1.0/1.0	512	400	0.5	0.8	0.47	QIR 4/4	Qr40
Dose 1.0 mGy	PCD-CT	Standard	120	13	1.0/1.0	512	400	0.5	0.8	1.03	QIR 4/4	Qr40
Dose 3.0 mGy	PCD-CT	Standard	120	38	1.0/1.0	512	400	0.5	0.8	3.00	QIR 4/4	Qr40
Dose 5.0 mGy	PCD-CT	Standard	120	63	1.0/1.0	512	400	0.5	0.8	4.98	QIR 4/4	Qr40
QIR level 0	PCD-CT	Standard	120	127	1.0/1.0	512	400	0.5	0.8	10.00	QIR 0/4	Qr40
QIR level 2	PCD-CT	Standard	120	127	1.0/1.0	512	400	0.5	0.8	10.00	QIR 2/4	Qr40
Kernel Qr44	PCD-CT	Standard	120	127	1.0/1.0	512	400	0.5	0.8	10.00	QIR 4/4	Qr44
Kernel Qr48	PCD-CT	Standard	120	127	1.0/1.0	512	400	0.5	0.8	10.00	QIR 4/4	Qr48

*PCD-CT* photon counting detector CT, *QIR* quantum iterative reconstruction

### Image segmentation

The segmentation was conducted by the same radiologist in our previous study with 6 years of experience in radiomics research [51]. The images were exported with Digital Imaging and Communications in Medicine (DICOM) format, converted to Neuroimaging Informatics Technology Initiative (NIFTI) using MRIcroGL software version 1.2.20220720b (https://www.nitrc.org/frs/?group_id=889), and loaded into ITK-SNAP software version 4.0.2 (http://www.itksnap.org/pmwiki/pmwiki.php) for analysis. The regions of interest (ROIs) were delineated by the radiologist in the same manner in our previous study [51], but not copied from the previous study as they are two independent parts of our series of studies. The ROIs of 35 pixels (approximately 27 mm for standard scans and 24 mm for high-pitch scans) were placed at the center of twenty-eight materials at the middle layer image from the reference scan, and then copied to other scans [33, 38–41, 51, 61]. The ROIs were used to generate ROIs for the middle twenty-five layers of images to allow reproducibility analysis.

### Feature extraction

The feature extraction was conducted by the same radiologist in our previous study with 6 years of experience in radiomics research [51]. We did not use any pre-processing steps before the feature extraction. The feature extraction was performed using Python version 3.12.1 (https://www.python.org) using PyRadiomics package version 3.0.1 (https://pyradiomics.readthedocs.io/en/latest/). As the ROIs were kept unchanged in the current study, the shape features were not extracted. The extracted features included 18 first-order, 24 gray-level co-occurrence matrix (GLCM), 14 gray-level run length matrix, 16 gray-level zone length matrix, 16 gray-level dependence matrix, and 5 neighborhood gray-tone difference matrix features [62]. The feature calculations and the settings of feature extraction are presented in Supplementary Note S2.

### Statistical analysis

The statistical analysis was performed by a radiologist with 6 years of experience in radiomics research using R language version 4.1.3 (https://www.r-project.org/) within RStudio version 1.4.1106 (https://posit.co/). As the test-retest repeatability has been found to be high in previous studies [51, 54], we focused on the reproducibility and variability of radiomics features in the current study. The reproducibility of features between protocols with changing influencing factors was evaluated using the intraclass correlation coefficient (ICC) [63] and concordance correlation coefficient (CCC) [64]. The variability of features due to changing influencing factors in different protocols was assessed by coefficient of variation (CV) [65] and quartile coefficient of dispersion (QCD) [66]. The variability of features was estimated across thirteen protocols for each of the twenty-eight materials by CV and QCD. The ICC and CCC values were interpreted as moderate (< 0.50), moderate (0.50–0.75), good (0.75–0.90), or excellent (≥ 0.90), while the CV and QCD values were interpreted as acceptable (< 10%), moderate but still adequate (11–20%) or too high and inadequate (≥ 20%) [63, 67, 68].

## Results

### Reproducibility of radiomics due to each influencing factor

**Fig. 3:**
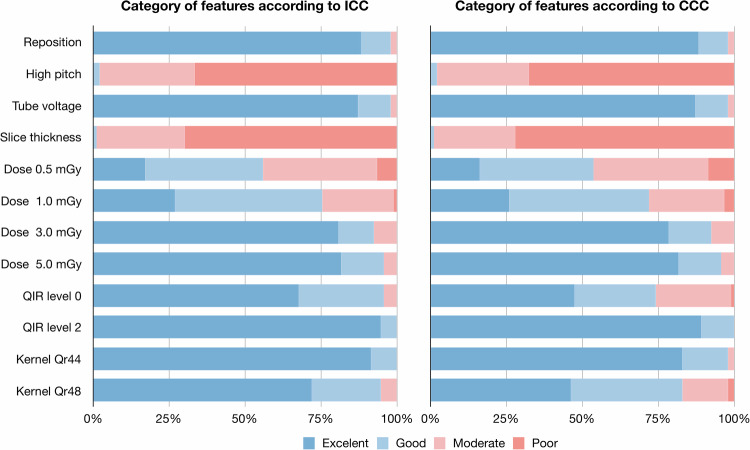
Percentage of robust radiomics features per influencing factor according to ICC and CCC. The percentage of robust features according to ICC and CCC values, in terms of repeatability, scanner, high-pitch scan, tube voltage, slice thickness/increment, radiation dose level, QIR level, and reconstruction kernel. The ICC and CCC values were interpreted as follows: poor, < 0.50; moderate, 0.50–0.75; good, 0.75–0.90; or excellent, ≥ 0.90

The mean ± standard deviation, median (interquartile range) of ICC and CCC values of reproducibility of radiomics due to each influencing factor were 0.255 ± 0.625, 0.146 (0.084, 0.244) and 0.189 ± 0.661, 0.102 (0.058, 0.173) (Table 2). The percentage of features with ICC > 0.90 and CCC > 0.90 were high when repositioned (88.2% and 88.2%) and tube voltage was changed (87.1% and 87.1%), but none of the features with ICC > 0.90 and CCC > 0.90 when high-pitch scan and different slice thickness were used (Fig. 3). The reproducibility of each radiomics feature according to influencing factors is presented (Supplementary Fig. S1). The radiomics features were robust to tube voltage, radiation dose, QIR strength level, and reconstruction kernel, but brittle to high-pitch scan and slice thickness.

**Table 2:** Reproducibility of radiomics due to each influencing factor

Influencing factor	ICC	CCC
Reposition	0.953 ± 0.061, 0.967 (0.950, 0.989)	0.953 ± 0.061, 0.967 (0.950, 0.989)
High-pitch scan	0.417 ± 0.191, 0.424 (0.285, 0.571)	0.405 ± 0.193, 0.398 (0.276, 0.548)
Tube voltage	0.943 ± 0.069, 0.960 (0.937, 0.982)	0.942 ± 0.070, 0.960 (0.936, 0.981)
Slice thickness	0.381 ± 0.171, 0.400 (0.239, 0.506)	0.378 ± 0.171, 0.396 (0.236, 0.504)
Dose 0.5 mGy	0.758 ± 0.151, 0.766 (0.686, 0.860)	0.741 ± 0.160, 0.763 (0.657, 0.844)
Dose 1.0 mGy	0.819 ± 0.128, 0.834 (0.756, 0.909)	0.810 ± 0.131, 0.831 (0.735, 0.907)
Dose 3.0 mGy	0.920 ± 0.079, 0.928 (0.906, 0.980)	0.918 ± 0.079, 0.927 (0.904, 0.980)
Dose 5.0 mGy	0.932 ± 0.073, 0.947 (0.927, 0.975)	0.931 ± 0.074, 0.947 (0.927, 0.975)
QIR level 0	0.910 ± 0.079, 0.923 (0.869, 0.964)	0.848 ± 0.134, 0.876 (0.734, 0.957)
QIR level 2	0.974 ± 0.032, 0.983 (0.969, 0.993)	0.956 ± 0.047, 0.976 (0.926, 0.993)
Kernel Qr44	0.965 ± 0.044, 0.978 (0.963, 0.985)	0.942 ± 0.068, 0.964 (0.927, 0.984)
Kernel Qr48	0.909 ± 0.085, 0.936 (0.898, 0.955)	0.852 ± 0.142, 0.893 (0.812, 0.948)

Data are presented as mean ± standard deviation, median (interquartile range)

*CCC* concordance correlation coefficient, *ICC* intraclass correlation coefficient, *QIR* quantum iterative reconstruction

### Variability of radiomics due to each influencing factor

**Fig. 4:**
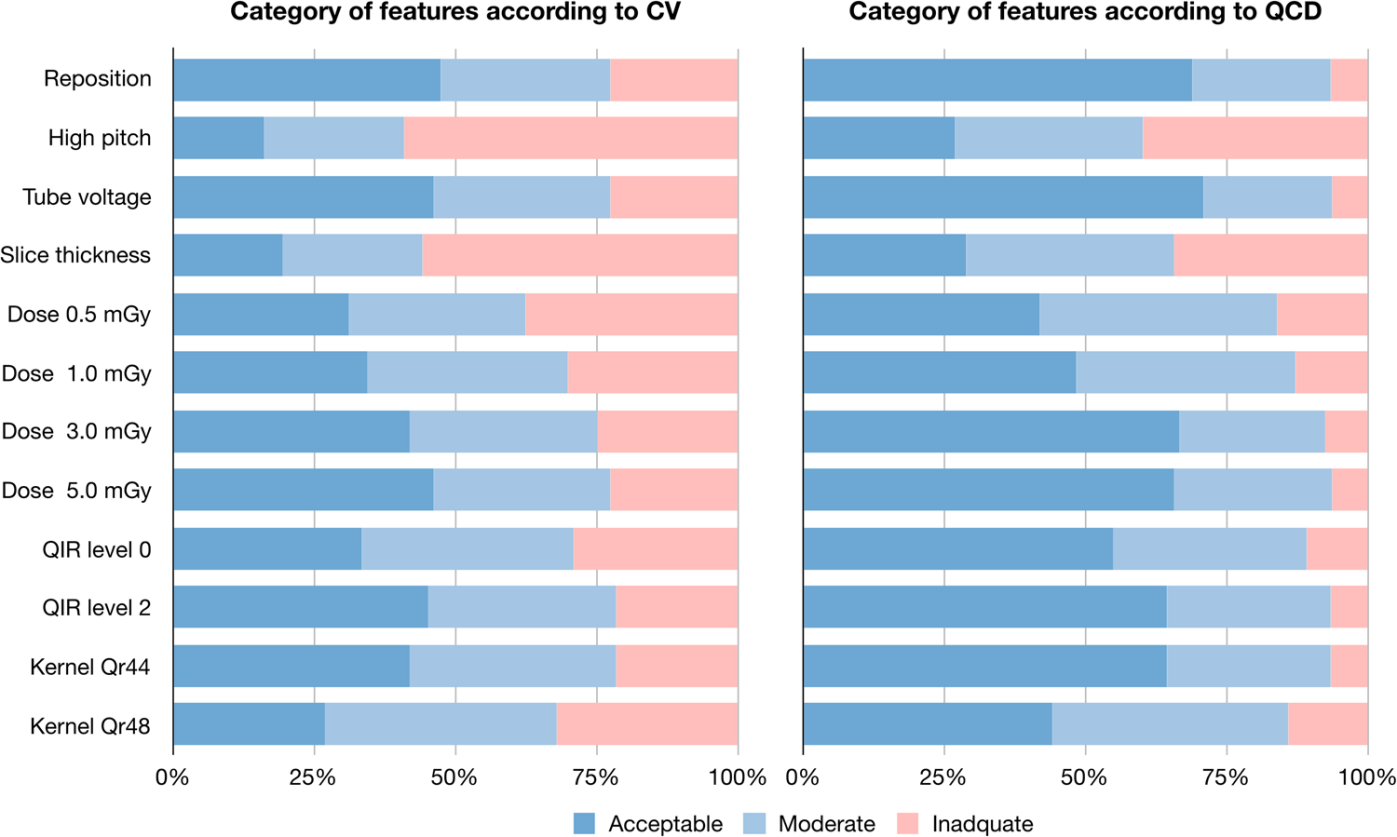
Percentage of robust radiomics features per influencing factor according to CV and QCD. The percentage of robust features according to CV and QCD values, in terms of repeatability, scanner, high-pitch scan, tube voltage, slice thickness/increment, radiation dose level, QIR level, and reconstruction kernel. The CV and QCD values were interpreted as follows: acceptable, < 10%; moderate but still adequate, 11–20%; and too high and inadequate, ≥ 20%

The mean ± standard deviation, median (interquartile range) of CV and QCD values of reproducibility of radiomics due to each influencing factor were 25.5% ± 62.5%, 14.6% (8.4%, 24.4%) and 18.9% ± 66.1%, 10.2% (5.8%, 17.3%) (Table 3). The percentage of features with CV < 10% and QCD < 10% were high when repositioned (47.3% and 68.8%) and tube voltage was changed (64.2% and 71.0%), but that with CV < 10% and QCD < 10% were low between standard and high-pitch scans (16.1% and 26.9%) and slice thickness (19.4% and 29.0%) (Fig. 4). The variability of each radiomics feature according to influencing factors is presented (Supplementary Fig. S2). The GLCM features were relatively less variable than other feature families, regardless of influencing factors.

**Table 3:** Variability of radiomics due to each influencing factor

Influencing factor	CV	QCD
Reposition	14.9% ± 13.9%, 11.0% (6.5%, 17.9%)	9.7% ± 12.1%, 7.0% (4.2%, 11%)
High-pitch scan	31.8% ± 33.3%, 22.6% (11.6%, 35.0%)	24.8% ± 31.1%, 18.0% (8.7%, 29.2%)
Tube voltage	15.3% ± 14.3%, 11.2% (7.2%, 18.3%)	9.3% ± 8.1%, 6.9% (4.4%, 10.9%)
Slice thickness	35.8% ± 59.2%, 22.0% (11.1%, 33.6%)	28.7% ± 54.9%, 16.6% (8.7%, 27.6%)
Dose 0.5 mGy	22.5% ± 26.7%, 16.7% (9.1%, 24.3%)	16.7% ± 29.5%, 11.4% (6.2%, 16.4%)
Dose 1.0 mGy	20.4% ± 21.3%, 14.3% (8.6%, 22.5%)	14.6% ± 23.8%, 10.4% (5.4%, 14.8%)
Dose 3.0 mGy	17.1% ± 17.4%, 11.9% (7.9%, 18.9%)	11.4% ± 14.6%, 7.6% (4.8%, 12.6%)
Dose 5.0 mGy	15.8% ± 15.1%, 11.4% (7.3%, 18.6%)	9.7% ± 8.6%, 7.4% (4.8%, 11.8%)
QIR level 0	17.9% ± 16.3%, 13.5% (8.4%, 21.3%)	12.1% ± 9.7%, 9.3% (6.1%, 14.7%)
QIR level 2	15.7% ± 14.3%, 11.7% (7.3%, 18.4%)	9.9% ± 8.1%, 7.7% (4.8%, 12.4%)
Kernel Qr44	16.3% ± 14.9%, 12.7% (8.0%, 18.7%)	11.3% ± 15.9%, 8.6% (5.1%, 12.5%)
Kernel Qr48	25.3% ± 67.5%, 15.1% (9.4%, 22.3%)	13.1% ± 10.3%, 11.3% (6.5%, 15%)

Data are presented as mean ± standard deviation, median (interquartile range)

*CV* coefficient of variation, *QCD* quartile coefficient of dispersion, *QIR* quantum iterative reconstruction

### Variability of radiomics according to each material

**Fig. 5:**
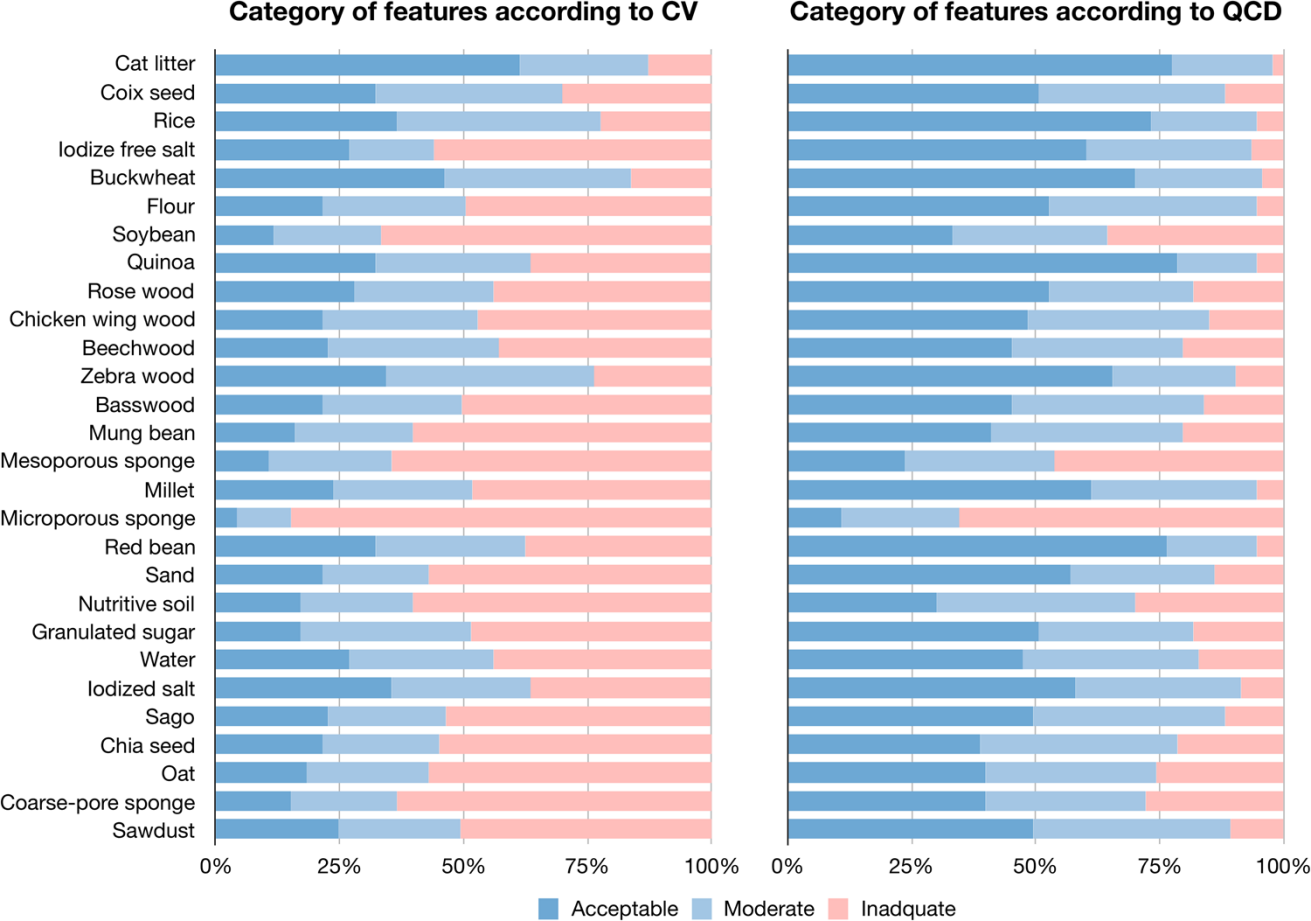
Percentage of robust radiomics features per ROI according to CV and QCD. The percentage of robust features according to CV and QCD values, in terms of repeatability, scanner, high-pitch scan, tube voltage, slice thickness/ increment, radiation dose level, QIR level, and reconstruction kernel. The CV and QCD values were interpreted as follows: acceptable, < 10%; moderate but still adequate, 11–20%; and too high and inadequate, ≥ 20%

The variability of features ranged largely across thirteen protocols for each of the twenty-eight materials (Table 4). The mean CV and QCD values ranged from 10.4% to 80.8%, and from 6.7% to 30.2%. The median CV and QCD values ranged from 8.6% to 29.9%, and from 5.7% to 27.4%. The percentage of features with CV < 10% were from 4.3% to 61.3%, and those with QCD < 10% were 10.8–78.5% among twenty-eight materials (Fig. 5). The variability of each radiomics feature according to materials is presented (Supplementary Fig. S3). The GLCM features were relatively less variable than other feature families, regardless of materials.

**Table 4:** Variability of radiomics according to each material

ROI	Material	CV	QCD
1	Cat litter	10.4% ± 8.2%, 8.6% (3.7%, 14.5%)	6.7% ± 5.2%, 5.7% (2.5%, 9.5%)
2	Coix seed	17.3% ± 14.3%, 14.8% (7.7%, 21.3%)	11.1% ± 9.1%, 10.0% (4.7%, 13.8%)
3	Rice	15.7% ± 14.8%, 12.3% (6.9%, 19.1%)	8.2% ± 6.5%, 6.9% (4.1%, 10.2%)
4	Iodize free salt	51.2% ± 97.0%, 20.8% (9.9%, 33.8%)	9.9% ± 7.7%, 8.7% (4.9%, 13%)
5	Buckwheat	12.9% ± 10.0%, 10.6% (5.8%, 16.6%)	8.3% ± 5.9%, 7.4% (3.5%, 10.8%)
6	Flour	39.0% ± 61.5%, 19.5% (11.9%, 33.8%)	10.9% ± 8.4%, 9.7% (5.8%, 13.7%)
7	Soybean	44.2% ± 64.1%, 29.9% (15.8%, 41.5%)	20.5% ± 28.7%, 14.2% (7.2%, 24.2%)
8	Quinoa	28.3% ± 46.4%, 12.8% (8.1%, 25.1%)	7.8% ± 5.8%, 7.0% (3.4%, 9.8%)
9	Rosewood	30.3% ± 39.7%, 17.9% (8.3%, 34.6%)	11.7% ± 10.7%, 8.4% (4.1%, 15.8%)
10	Chicken wing wood	30.9% ± 41.1%, 17.8% (11.7%, 29.3%)	13.2% ± 13.1%, 10.1% (5.4%, 16.8%)
11	Beechwood	22.1% ± 18.0%, 17.0% (11.8%, 28.7%)	13.3% ± 10.5%, 10.6% (5.6%, 18.7%)
12	Zebra wood	19.6% ± 35.6%, 13.9% (8.6%, 19.2%)	11.1% ± 19.1%, 6.7% (4.0%, 12.4%)
13	Basswood	31.7% ± 42.1%, 20.8% (11.4%, 31.1%)	14.4% ± 13.6%, 11.7% (5.2%, 18.9%)
14	Mung bean	39.0% ± 53.8%, 26.1% (13.7%, 38.7%)	18.4% ± 38.3%, 12.2% (6.6%, 18.1%)
15	Mesoporous sponge	51.0% ± 69.0%, 29.1% (15.2%, 40.4%)	23.8% ± 29.9%, 18.8% (10.2%, 26.5%)
16	Millet	33.2% ± 56.8%, 18.8% (10.6%, 31.6%)	10.9% ± 12.8%, 8.1% (4.6%, 11.7%)
17	Microporous sponge	55.8% ± 48.2%, 41.8% (27.4%, 62.5%)	30.2% ± 19.2%, 27.4% (15.6%, 41.0%)
18	Red bean	35.6% ± 61.9%, 13.2% (8.1%, 27.9%)	8.0% ± 6.9%, 6.7% (3.6%, 9.8%)
19	Sand	49.2% ± 74.3%, 23.6% (11.6%, 38.0%)	13.3% ± 18.5%, 9.1% (3.7%, 14.3%)
20	Nutritive soil	39.6% ± 48.9%, 23.2% (11.4%, 41.2%)	19.9% ± 27.0%, 15.5% (7.8%, 20.9%)
21	Granulated sugar	25.8% ± 23.3%, 19.6% (12.0%, 30.3%)	12.2% ± 10.6%, 9.9% (4.3%, 17.4%)
22	Water	20.9% ± 18.1%, 16.6% (9.3%, 27.3%)	13.6% ± 11.7%, 10.7% (6.1%, 17.4%)
23	Iodized salt	28.1% ± 51.5%, 16.1% (7.3%, 25.6%)	11.2% ± 12.5%, 8.7% (3.7%, 15.0%)
24	Sago	27.1% ± 30.3%, 20.3% (10.4%, 28.0%)	12.3% ± 10.9%, 10.1% (5.9%, 14.4%)
25	Chia seed	80.8% ± 51.3%, 21.2% (10.9%, 42.2%)	18.0% ± 26.5%, 12.0% (5.9%, 18.0%)
26	Oat	26.9% ± 25.0%, 24.4% (12.2%, 29.6%)	15.7% ± 14.7%, 12.2% (6.7%, 20.2%)
27	Coarse-pore sponge	44.4% ± 55.8%, 24.9% (14.0%, 46.6%)	17.0% ± 16.3%, 13.3% (7.2%, 21.9%)
28	Sawdust	33.8% ± 44.4%, 20.1% (10.0%, 31.8%)	11.6% ± 9.8%, 10.5% (5.3%, 14.3%)

Data are presented as mean ± standard deviation, median (interquartile range)

*CV* coefficient of variation, *QCD* quartile coefficient of dispersion, *QIR* quantum iterative reconstruction, *ROI* region of interest

## Discussion

In this study, we demonstrated that the radiomics features within PCD-CT were brittle to high-pitch scan and slice thickness that influenced voxel size. In contrast, the radiomics features were relatively robust against repositioning, tube voltage, radiation dose level, QIR strength level, and reconstruction kernels within PCD-CT. The high-pitch scan and slice thickness should be set with careful attention within PCD-CT to allow higher robustness of radiomics features before the implementation of radiomics analysis in clinical routine.

The high-pitch scan and slice thickness would severely damage the robustness of the radiomics features. The slice thickness can directly change the voxel size in the radiomics analysis [28, 33, 34]. With no doubt, it can also significantly change the radiomics feature values within the PCD-CT system, as the voxel in the scans with different slice thicknesses corresponds to the different physical parts of the object. In contrast, it has been demonstrated that the pitch has a limited impact on the stability of the radiomics features [69]. Therefore, we supposed that the main source of difference between the reference scan and high-pitch scan was the field of view (FOV) which can also directly change the voxel size. We did not make the FOV the same in the reference scan and the high-pitch scan, but used their default settings to show the influence of standard and high-pitch scans. The results indicated that careful harmonization between standard and high-pitch scans is necessary within PCD-CT systems to allow higher robustness of radiomics features.

The results of our reposition test were in accordance with the test-retest radiomics stability analysis in the PCD-CT using organic phantoms and patient data [54, 55], showing the high repeatability of the PCD-CT radiomics features. It is necessary to investigate the influence of different tube voltages. A recent consensus paper recommended 140 kVp for abdominal scans that challenged the recommendation by the manufacturer of 120 kVp [70], as it is believed that 140 kVp can optimize the material spectral separation in the abdomen. In EID-CT, the tube voltage of the scans has a small impact on the radiomics feature values, because the difference in CT values was relatively small in the exported polychromatic images acquired at 120 kVp and 140 kVp [33, 34]. On the other hand, the stability of radiomics features in PCD-CT benefited from the default setting of exporting all the VMI at the same energy level of 70 keV regardless of the tube voltage [70]. Dunning et al [50] suggested that the change of radiomics feature values between 10 mGy and 60 mGy scans were both small within EID-CT and PCD-CT systems. However, the radiation dose level used in this phantom study was relatively high and impossible in the patient scans. Hertel et al [54] performed a similar phantom study using a tube voltage of 120 kVp with a tube current of 10 mAs, 50 mAs, and 100 mAs. It indicated that the PCD-CT system can provide highly stable radiomics feature values with a much lower dose level. Our study expanded their studies by comparing five radiation dose levels, and showed that the PCD-CT system is still robust for radiomics feature values at a dose level that was recently available in clinical practice [71–73]. As the PCD-CT can escape from the background electronic noise [74], it is reasonable that PCD-CT can provide high radiomics feature reproducibility between different radiation dose levels [50, 51, 54].

The reconstruction algorithms and strength levels of the reconstruction algorithms were considered to have a great impact on the radiomics stability [38–41, 75–77]. However, we did not identify the obvious change in radiomics feature values due to the strength level of QIR. The high levels of QIR have been confirmed to have the ability to reduce image noise and improve lesion conspicuity without compromising image texture or CT attenuation values [78]. Thus, the impact on the radiomics features of QIR that are specially developed for PCD-CT systems is expected to be less than the iteration reconstruction algorithms or deep learning-based image reconstruction algorithms in EID-CT systems. The reconstruction kernels were also recognized as an important source of variability of radiomics feature values, because they can severely change the image texture [75, 79]. Surprisingly, the Qr40, Qr44, and Qr48 kernels that were compared in our study did not show a significant impact on the radiomics features. We supposed that the conservative setting of the kernel can purtily explain the phenomenon. Nevertheless, the setting used in our study is reasonable as the reconstruction kernels used for the same disease or those occurring in a specific body part may be close to each other.

Our study has several limitations. First, this was a phantom study without validation in human participants. As repeated scans are not available among human participants, it is necessary to test the influence of the acquisition parameters using a phantom. Our texture phantom has the advantage of richer texture and more various densities than those homogeneous phantoms, to better present the physiological human parenchyma or pathological tissues [38–41]. Further, the organic phantoms with pathological changes may be preferred to give deeper insight into the radiomics features derived from a specific disease [80, 81]. Moreover, our conclusion should be validated using human data to present the radiomics reproducibility in actual physiological phenomena or true pathological tissues. Second, only one PCD-CT system from a specific manufacturer was used in the study. It is unclear whether results in this PCD-CT system can be directly transferred to other PCD-CT systems. Further, it would be interesting to compare the robustness of radiomics features among different PCD-CT systems in the future. The PCD-CT systems were expected to better present the true inner structure of the object with lower image noise [50–53]. It would be exciting to find out whether the difference among PCD-CT techniques is smaller than that among EID-CT systems. Third, we did not strictly register the FOV between the reference scan and high-pitch scans, as the limited FOV of the high-pitch scan and the default FOV are different. Our study indicated that the radiomics models based on default scans cannot be directly transferred to high-pitch scans. However, the true influence of the pitch and rotation time was not evaluated. It is necessary to test the influence of pitch with other parameters unchanged or after a post-process step of resampling. Although the high-pitch may negatively affect the image quality and potentially change the radiomics features, it is desirable for fast imaging [82–84]. Fourth, there are more potentially influencing factors that can be investigated. We are planning to test the impact factors that are unique in the PCD-CT system on the radiomics features, such as energy threshold, energy bins, detector pixel size, detector pixel configuration, X-ray focal spot, scan mode, detector operation mode, collimation, etc. An in-depth investigation of these parameters on radiomics feature stability is warranted before the radiomics analysis to provide a scientific basis for it. Fifth, the influence of segmentation was not investigated in our study. Segmentation can be an important source of variations in radiomics analysis [85]. However, it is hard to be a source of variation in our study because we acquired all the images with the position unchanged and used the same segmentation in all the scans to avoid the variations due to the segmentation as the previous studies did [33, 38–41, 51]. It is also out of our study's aim to investigate the influence of acquisition and reconstruction parameters on radiomics robustness within PCD-CT. Nevertheless, we do consider segmentation is an important topic in radiomics analysis, and will investigate this point in the future. Finally, the impact of robustness on the clinical radiomics analysis was not evaluated. The performance of radiomics models established using different CT systems should be assessed. It is expected that the PCD-CT systems can better characterize disease with radiomics features of richer textures and smaller volumes [45–49]. Whether it can translate into greater model performance than the EID-CT systems is of interest.

To conclude, this study evaluated the acquisition and reconstruction parameters on the robustness of radiomics features, and presented their varying degree of influence within the PCD-CT system. The parameters that changed the voxel size had a greater impact on the robustness of radiomics features than those that did not. Further studies are desired to optimize the imaging protocols in PCD-CT system to guarantee the radiomic feature stability before the radiomics analysis.

## Supplementary information

ELECTRONIC SUPPLEMENTARY MATERIAL

## Abbreviations

CCC: Concordance correlation coefficient
CV: Coefficient of variation
EID-CT: Energy-integrating detector CT
FOV: Field of view
ICC: Intraclass correlation coefficient
PCD-CT: Photon-counting detector CT
QCD: Quartile coefficient of dispersion
QIR: Quantum iterative reconstruction
ROI: Region of interest
VMI: Virtual monochromatic image
