# Supplementary material for: Robustness of radiomics within photon-counting detector CT: impact of acquisition and reconstruction factors
Source: Eur Radiol. 2025 Jan 31;35(8):4661–73. doi: 10.1007/s00330-025-11374-x (PMC12226652; doi:10.1007/s00330-025-11374-x)
Supplement: Supplementary file 1 — ELECTRONIC SUPPLEMENTARY MATERIAL [file 330_2025_11374_MOESM1_ESM.pdf]

Robustness of radiomics within photon-counting detector CT: impact of acquisition and reconstruction factors
ELECTRONIC SUPPLEMENTARY MATERIAL

List of Supplementary Materials

Supplementary Note S1 Details of the set-ups of texture phantom
Supplementary Note S2 Radiomics feature extraction method

Supplementary Figure S1 Heatmap of reproducibility of radiomics features per influencing factor
Supplementary Figure S2 Heatmap of variability of radiomics features per influencing factor
Supplementary Figure S3 Heatmap of variability of radiomics features according to materials

Eur Radiol (2025) Zhang H, Lu T, Wang L, et al.

Supplementary Note 1 Details of the set-ups of texture phantom

We established a texture phantom consisting of twenty-eight different materials. There were five wood blocks and twenty-three bottles filled with different materials.

(1) Wood blocks

The wood block was cuboid with a size of 150 mm × 55 mm × 45 mm. We bought and asked the seller to cut the wood into the size of 150 mm × 55 mm × 45 mm. The types of wood were selected to present different textures. We ask the seller to recommend the types of woods with heterogenous densities and textures. He kindly recommended the following five types of wood: rose wood, chicken wing wood, beechwood, zebra wood, and basswood.

(2) Bottles filled with different materials

We recycled the bottles of juice bought in the convenience store. Our colleagues drank the juice, and cleaned the bottles using fresh water. The bottles were then naturally dried for this study. They were also asked to provide various materials with heterogenous densities and textures.

The materials that they brought from their home or we bought from the supermarket or online includes following: mesoporous sponge, iodize free salt, granulated sugar, flour, iodized salt, coarse-pore sponge, nutritive soil for succulent plants, sand, microporous sponge, coix seed, buckwheat, sago, cat litter, oat, sawdust, soybean, red bean, mung bean, rice, quinoa, millet, and chia seed. We additionally included a bottle filled with air.

The cuboid part of the bottle was with a size of 130 mm × 55 mm × 45 mm. The cuboid part bottle was filled with materials as tightly as possible, and tissue was put into the cylindrical part of the bottle if needed to avoid unexpected movement. These materials were selected to give us varying texture. The materials were positioned to avoid beam-hardening artifacts and were kept unchanged throughout all the scans in the study.

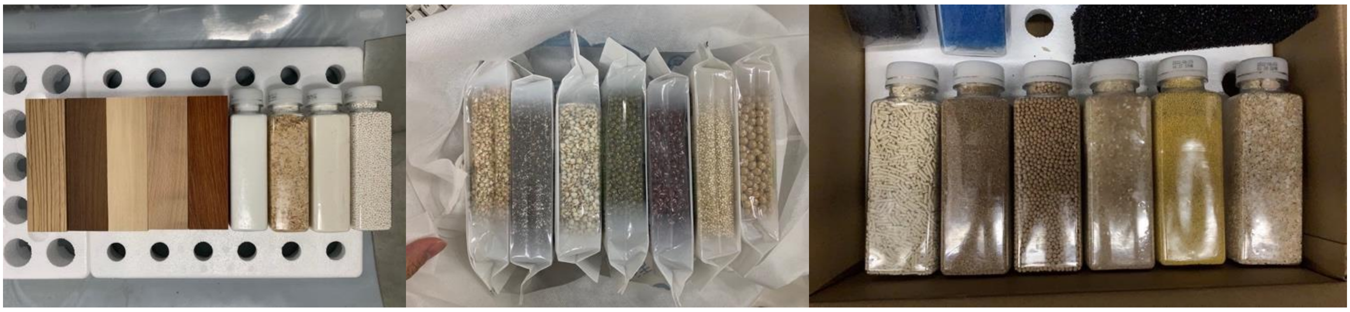

Supplementary Note 2 Radiomics feature extraction method

(1) yaml document for radiomics feature extraction

We did not perform any post-processing steps (such as resampling, normalization, combat, etc.) before the feature extraction.


```
featureClass:
  firstorder: null
  glcm: null
  gldm: null
  glrlm: null
  glszm: null
  ngtdm: null
imageType:
  Original: {}
setting:
  additionalInfo: false
  binCount: 16
  force2D: true
  geometryTolerance: 1e-6
  label: 1
  resegmentMode: sigma
```


(2) Name of calculated features

The details of calculation of each feature are available via the website of PyRadiomics (https://pyradiomics.readthedocs.io/en/latest/).

No	Feature famility	Name of features
1	firstorder	10Percentile
2	firstorder	90Percentile
3	firstorder	Energy
4	firstorder	Entropy
5	firstorder	InterquartileRange
6	firstorder	Kurtosis
7	firstorder	Maximum
8	firstorder	MeanAbsoluteDeviation
9	firstorder	Mean
10	firstorder	Median
11	firstorder	Minimum
12	firstorder	Range
13	firstorder	RobustMeanAbsoluteDeviation
14	firstorder	RootMeanSquared
15	firstorder	Skewness
16	firstorder	TotalEnergy
17	firstorder	Uniformity
18	firstorder	Variance
19	glcm	Autocorrelation
20	glcm	ClusterProminence
21	glcm	ClusterShade

22	glcm	ClusterTendency
23	glcm	Contrast
24	glcm	Correlation
25	glcm	DifferenceAverage
26	glcm	DifferenceEntropy
27	glcm	DifferenceVariance
28	glcm	Id
29	glcm	Idm
30	glcm	Idmn
31	glcm	Idn
32	glcm	Imc1
33	glcm	Imc2
34	glcm	InverseVariance
35	glcm	JointAverage
36	glcm	JointEnergy
37	glcm	JointEntropy
38	glcm	MCC
39	glcm	MaximumProbability
40	glcm	SumAverage
41	glcm	SumEntropy
42	glcm	SumSquares
43	gldm	DependenceEntropy
44	gldm	DependenceNonUniformity
45	gldm	DependenceNonUniformityNormalized
46	gldm	DependenceVariance
47	gldm	GrayLevelNonUniformity
48	gldm	GrayLevelVariance
49	gldm	HighGrayLevelEmphasis
50	gldm	LargeDependenceEmphasis
51	gldm	LargeDependenceHighGrayLevelEmphasis
52	gldm	LargeDependenceLowGrayLevelEmphasis
53	gldm	LowGrayLevelEmphasis
54	gldm	SmallDependenceEmphasis
55	gldm	SmallDependenceHighGrayLevelEmphasis
56	gldm	SmallDependenceLowGrayLevelEmphasis
57	glrlm	GrayLevelNonUniformity
58	glrlm	GrayLevelNonUniformityNormalized
59	glrlm	GrayLevelVariance
60	glrlm	HighGrayLevelRunEmphasis
61	glrlm	LongRunEmphasis
62	glrlm	LongRunHighGrayLevelEmphasis
63	glrlm	LongRunLowGrayLevelEmphasis
64	glrlm	LowGrayLevelRunEmphasis
65	glrlm	RunEntropy

66	glrlm	RunLengthNonUniformity
67	glrlm	RunLengthNonUniformityNormalized
68	glrlm	RunPercentage
69	glrlm	RunVariance
70	glrlm	ShortRunEmphasis
71	glrlm	ShortRunHighGrayLevelEmphasis
72	glrlm	ShortRunLowGrayLevelEmphasis
73	glszm	GrayLevelNonUniformity
74	glszm	GrayLevelNonUniformityNormalized
75	glszm	GrayLevelVariance
76	glszm	HighGrayLevelZoneEmphasis
77	glszm	LargeAreaEmphasis
78	glszm	LargeAreaHighGrayLevelEmphasis
79	glszm	LargeAreaLowGrayLevelEmphasis
80	glszm	LowGrayLevelZoneEmphasis
81	glszm	SizeZoneNonUniformity
82	glszm	SizeZoneNonUniformityNormalized
83	glszm	SmallAreaEmphasis
84	glszm	SmallAreaHighGrayLevelEmphasis
85	glszm	SmallAreaLowGrayLevelEmphasis
86	glszm	ZoneEntropy
87	glszm	ZonePercentage
88	glszm	ZoneVariance
89	ngtdm	Busyness
90	ngtdm	Coarseness
91	ngtdm	Complexity
92	ngtdm	Contrast
93	ngtdm	Strength

Supplementary Figure S1 Heatmap of reproducibility of radiomics features per influencing factor

(A) Heatmap of reproducibility of radiomics features per influencing factor by ICC

Family	Feature	Reposition	High pitch	Tube voltage	Slice thickness	Dose 0.5 mGy	Dose 1.0 mGy	Dose 3.0 mGy	Dose 5.0 mGy	QIR level 0	QIR level 2	Kernel Qr44	Kernel Qr48
firstorder	10Percentile	0.99997	0.21403	0.99991	0.56341	0.99507	0.99789	0.99969	0.99988	0.99994	0.99998	0.99951	0.99764
firstorder	90Percentile	0.99999	0.42402	0.99993	0.48122	0.99660	0.99855	0.99979	0.99994	0.99995	0.99999	0.99978	0.99905
firstorder	Energy	1.00000	0.28502	0.99978	0.26844	0.99900	0.99944	0.99989	0.99996	0.99999	1.00000	0.99988	0.99942
firstorder	Entropy	0.95538	0.54434	0.95956	0.55606	0.76187	0.84957	0.91963	0.93720	0.95520	0.99177	0.98068	0.93931
firstorder	InterquartileRange	0.99978	0.70716	0.99957	0.50573	0.94305	0.97897	0.99744	0.99918	0.99937	0.99986	0.99561	0.98169
firstorder	Kurtosis	0.99760	0.42453	0.99804	0.39269	0.87156	0.95957	0.99401	0.99744	0.94652	0.99312	0.96878	0.86672
firstorder	Maximum	0.99938	0.46223	0.99914	0.48206	0.99023	0.99550	0.99855	0.99893	0.99971	0.99996	0.99798	0.99165
firstorder	MeanAbsoluteDeviation	0.99996	0.65451	0.99977	0.46380	0.94749	0.98072	0.99783	0.99944	0.99968	0.99995	0.99664	0.98515
firstorder	Mean	1.00000	0.31096	0.99996	0.50791	0.99996	0.99996	0.99998	0.99999	1.00000	1.00000	1.00000	1.00000
firstorder	Median	0.99998	0.32654	0.99995	0.48941	0.99979	0.99987	0.99995	0.99997	0.99998	0.99999	0.99995	0.99977
firstorder	Minimum	0.99928	0.15926	0.99902	0.52727	0.98442	0.99240	0.99773	0.99841	0.99967	0.99995	0.99901	0.99595
firstorder	Range	0.99832	0.48189	0.99789	0.35606	0.95006	0.97853	0.99468	0.99649	0.99918	0.99987	0.99576	0.98268
firstorder	RobustMeanAbsoluteDeviation	0.99992	0.69456	0.99975	0.49732	0.94404	0.97940	0.99767	0.99937	0.99958	0.99993	0.99604	0.98258
firstorder	RootMeanSquared	1.00000	0.20431	0.99990	0.26869	0.99722	0.99876	0.99982	0.99995	0.99996	1.00000	0.99969	0.99856
firstorder	Skewness	0.99464	0.60996	0.98845	0.55608	0.89024	0.95524	0.98851	0.99237	0.96299	0.99489	0.99150	0.95803
firstorder	TotalEnergy	1.00000	0.28098	0.99978	0.18402	0.99900	0.99944	0.99989	0.99996	0.99999	1.00000	0.99988	0.99942
firstorder	Uniformity	0.96517	0.57639	0.96645	0.55973	0.79130	0.87915	0.93951	0.94715	0.94887	0.99069	0.98089	0.93810
firstorder	Variance	0.99995	0.71146	0.99971	0.56345	0.90075	0.95984	0.99534	0.99882	0.99944	0.99994	0.99088	0.96164
glcm	Autocorrelation	0.94716	0.45972	0.93415	0.45386	0.76404	0.84193	0.91461	0.92799	0.97108	0.99447	0.97935	0.95271
glcm	ClusterProminence	0.98302	0.82678	0.96956	0.76228	0.84819	0.90542	0.96398	0.97293	0.95874	0.98674	0.98535	0.95551
glcm	ClusterShade	0.98929	0.81726	0.90589	0.49725	0.86917	0.92376	0.97138	0.97760	0.94356	0.98380	0.98722	0.96495
glcm	ClusterTendency	0.97796	0.74919	0.97046	0.69197	0.83277	0.89630	0.95677	0.96722	0.95282	0.98794	0.98570	0.95484
glcm	Contrast	0.96603	0.27324	0.96135	0.15887	0.65186	0.77135	0.90459	0.94090	0.92859	0.98551	0.97595	0.91169
glcm	Correlation	0.99299	0.48509	0.98419	0.25584	0.85138	0.91597	0.97366	0.98753	0.91616	0.97965	0.96330	0.88509
glcm	DifferenceAverage	0.96862	0.33859	0.96281	0.24495	0.68717	0.79518	0.91521	0.94777	0.92122	0.98344	0.98314	0.94354
glcm	DifferenceEntropy	0.96778	0.43320	0.96177	0.23421	0.75849	0.84350	0.92754	0.94979	0.90200	0.98017	0.98455	0.95219
glcm	DifferenceVariance	0.96312	0.36103	0.95865	0.16920	0.68887	0.80021	0.90792	0.94034	0.90191	0.97964	0.97525	0.90841
glcm	Id	0.96913	0.39640	0.96014	0.34192	0.70584	0.80485	0.91783	0.94852	0.89938	0.97649	0.98214	0.94755
glcm	Idm	0.96933	0.38210	0.96143	0.31626	0.70326	0.80449	0.91836	0.94939	0.90513	0.97873	0.98290	0.94802
glcm	Idmn	0.96701	0.28648	0.96262	0.17134	0.66199	0.77919	0.90835	0.94360	0.92883	0.98568	0.97804	0.92072
glcm	Idn	0.96901	0.35547	0.96253	0.27117	0.69401	0.79937	0.91681	0.94868	0.91639	0.98201	0.98360	0.94721
glcm	Imc1	0.99529	0.61687	0.96893	0.12615	0.80344	0.88416	0.96432	0.98486	0.80694	0.95854	0.97161	0.91139
glcm	Imc2	0.99194	0.44657	0.98627	0.29805	0.86003	0.92085	0.97312	0.98577	0.91155	0.97796	0.96055	0.89346
glcm	InverseVariance	0.95634	0.57896	0.93372	0.50863	0.74505	0.81242	0.89539	0.92317	0.73852	0.91730	0.92675	0.81250
glcm	JointAverage	0.94925	0.51442	0.93239	0.50440	0.78702	0.85937	0.92231	0.93040	0.97304	0.99486	0.98143	0.95711
glcm	JointEnergy	0.97334	0.57083	0.97103	0.52532	0.82298	0.90937	0.95999	0.95329	0.92052	0.98592	0.98263	0.94506
glcm	JointEntropy	0.95258	0.45952	0.95420	0.50055	0.74275	0.83324	0.91125	0.93230	0.94722	0.98915	0.98169	0.94385
glcm	MCC	0.99144	0.46853	0.98009	0.24477	0.85993	0.92091	0.97382	0.98561	0.91508	0.98035	0.96572	0.91127
glcm	MaximumProbability	0.94695	0.54908	0.93442	0.55395	0.73211	0.84019	0.90595	0.92587	0.91286	0.97694	0.96485	0.92222
glcm	SumAverage	0.94925	0.51442	0.93239	0.50440	0.78702	0.85937	0.92231	0.93040	0.97304	0.99486	0.98143	0.95711
glcm	SumEntropy	0.96090	0.58326	0.96452	0.55513	0.79600	0.87400	0.93302	0.94470	0.96359	0.99305	0.98146	0.94087
glcm	SumSquares	0.97379	0.69494	0.96917	0.66586	0.81121	0.87961	0.94702	0.96127	0.95829	0.98971	0.98401	0.94796
gldm	DependenceEntropy	0.96803	0.57936	0.96527	0.45205	0.80629	0.88356	0.95042	0.95076	0.91310	0.98167	0.98057	0.94225
gldm	DependenceNonUniformity	0.96278	0.10433	0.94789	0.18051	0.62796	0.73744	0.93200	0.89292	0.80688	0.95430	0.96302	0.89786
gldm	DependenceNonUniformityNormalizec	0.96278	0.10433	0.94789	0.18051	0.62796	0.73744	0.93200	0.89292	0.80688	0.95430	0.96302	0.89786
gldm	DependenceVariance	0.94609	0.37932	0.93279	0.58801	0.52148	0.67431	0.86617	0.91085	0.86851	0.95227	0.95723	0.90610
gldm	GrayLevelNonUniformity	0.96517	0.57639	0.96645	0.55973	0.79130	0.87915	0.93951	0.94715	0.94887	0.99069	0.98089	0.93810
gldm	GrayLevelVariance	0.97365	0.69676	0.96841	0.66766	0.80957	0.87880	0.94690	0.96107	0.95750	0.98952	0.98388	0.94757
gldm	HighGrayLevelEmphasis	0.94976	0.46607	0.93732	0.45330	0.77251	0.84884	0.91857	0.93122	0.97244	0.99473	0.98057	0.95562
gldm	LargeDependenceEmphasis	0.96870	0.47949	0.95521	0.46022	0.73714	0.82376	0.92097	0.94641	0.85917	0.96249	0.97555	0.93598
gldm	LargeDependenceHighGrayLevelEmpha	0.93706	0.51182	0.94122	0.59863	0.52244	0.67408	0.86832	0.92346	0.84573	0.95951	0.96283	0.91293
gldm	LargeDependenceLowGrayLevelEmphas	0.98061	0.64378	0.97263	0.51935	0.85293	0.94992	0.98527	0.97238	0.93715	0.99088	0.99451	0.96933
gldm	LowGrayLevelEmphasis	0.96515	0.71928	0.95409	0.63378	0.85992	0.92871	0.96220	0.95531	0.97013	0.99442	0.98854	0.95814
gldm	SmallDependenceEmphasis	0.96052	0.26349	0.95425	0.23897	0.63353	0.73993	0.89131	0.93450	0.91828	0.97962	0.96987	0.91304
gldm	SmallDependenceHighGrayLevelEmpha	0.95649	0.32726	0.94606	0.24367	0.68607	0.78790	0.90574	0.93040	0.94808	0.98640	0.97226	0.92049
gldm	SmallDependenceLowGrayLevelEmphas	0.69062	0.09878	0.64326	0.25409	0.32757	0.42514	0.58812	0.60181	0.81581	0.93146	0.88255	0.71031
glrlm	GrayLevelNonUniformity	0.97383	0.61528	0.95479	0.36067	0.78896	0.87885	0.94140	0.95591	0.85195	0.96342	0.95350	0.84830
glrlm	GrayLevelNonUniformityNormalized	0.96081	0.54220	0.96152	0.56043	0.78473	0.85850	0.92822	0.94356	0.95726	0.99257	0.98064	0.93720
glrlm	GrayLevelVariance	0.95996	0.64469	0.94983	0.59872	0.76582	0.84671	0.92344	0.94138	0.94181	0.98539	0.97644	0.92626
glrlm	HighGrayLevelRunEmphasis	0.93886	0.41480	0.92233	0.44919	0.75103	0.83112	0.90245	0.91582	0.97153	0.99433	0.97610	0.94559
glrlm	LongRunEmphasis	0.99139	0.11129	0.98241	0.09488	0.84571	0.73231	0.98836	0.95438	0.92320	0.98525	0.97442	0.89494
glrlm	LongRunHighGrayLevelEmphasis	0.91157	0.41123	0.91647	0.43587	0.41442	0.53621	0.81777	0.88695	0.70156	0.89898	0.93204	0.84144
glrlm	LongRunLowGrayLevelEmphasis	0.99194	0.08081	0.98304	0.04639	0.84146	0.73157	0.98957	0.95469	0.92336	0.98572	0.97582	0.89754
glrlm	LowGrayLevelRunEmphasis	0.96425	0.74384	0.95107	0.67702	0.87175	0.92324	0.95619	0.94942	0.97077	0.99441	0.98613	0.96196
glrlm	RunEntropy	0.98795	0.58975	0.95380	0.17631	0.74205	0.83417	0.94086	0.96814	0.76325	0.93942	0.95529	0.85048
glrlm	RunLengthNonUniformity	0.96466	0.32896	0.95595	0.33898	0.65779	0.76455	0.90052	0.94113	0.89813	0.97578	0.97761	0.93275
glrlm	RunLengthNonUniformityNormalized	0.96531	0.39232	0.95567	0.36545	0.68639	0.78206	0.90562	0.94286	0.88591	0.97199	0.97637	0.93281
glrlm	RunPercentage	0.96842	0.43561	0.95644	0.41227	0.71453	0.80724	0.91644	0.94630	0.87694	0.96871	0.97764	0.93838
glrlm	RunVariance	0.97968	0.23686	0.95642	0.17815	0.78409	0.86489	0.98219	0.86670	0.84522	0.96778	0.96758	0.89294
glrlm	ShortRunEmphasis	0.96556	0.44965	0.95457	0.39308	0.72086	0.80149	0.91251	0.94452	0.86208	0.96443	0.97146	0.92549
glrlm	ShortRunHighGrayLevelEmphasis	0.94694	0.38585	0.92633	0.37842	0.76483	0.84114	0.90858	0.92067	0.96990	0.99233	0.97700	0.94344
glrlm	ShortRunLowGrayLevelEmphasis	0.85439	0.42042	0.82219	0.48445	0.66503	0.75601	0.84108	0.82078	0.92760	0.97945	0.94887	0.86319
glszm	GrayLevelNonUniformity	0.96853	0.27946	0.96148	0.15772	0.71007	0.80303	0.92584	0.95396	0.90769	0.97655	0.96518	0.90424
glszm	GrayLevelNonUniformityNormalized	0.90779	0.64286	0.93146	0.50522	0.71266	0.84553	0.90783	0.92739	0.92811	0.97869	0.93924	0.85655
glszm	GrayLevelVariance	0.78484	0.52026	0.75985	0.24617	0.42821	0.58341	0.69729	0.72398	0.77041	0.90021	0.87178	0.76028
glszm	HighGrayLevelZoneEmphasis	0.89010	0.34352	0.85459	0.44326	0.68683	0.75620	0.83626	0.84565	0.95567	0.98629	0.95542	0.90494
glszm	LargeAreaEmphasis	0.98902	0.25754	0.98295	0.20094	0.90469	0.69736	0.98140	0.97484	0.85245	0.97712	0.98367	0.93792
glszm	LargeAreaHighGrayLevelEmphasis	0.82792	0.07788	0.81037	0.23981	0.33391	0.53785	0.74033	0.82101	0.52560	0.82805	0.85226	0.75684
glszm	LargeAreaLowGrayLevelEmphasis	0.98918	0.16182	0.98237	0.10198	0.88477	0.67233	0.98565	0.97686	0.84898	0.97825	0.98502	0.93606
glszm	LowGrayLevelZoneEmphasis	0.86346	0.46511	0.81808	0.48346	0.73743	0.79872	0.86497	0.84212	0.92792	0.98281	0.94413	0.82313
glszm	SizeZoneNonUniformity	0.94129	0.09477	0.93763	0.14547	0.52815	0.63544	0.83930	0.89927	0.90281	0.96861	0.93040	0.82212
glszm	SizeZoneNonUniformityNormalized	0.83990	0.04757	0.85535	0.17847	0.44285	0.52314	0.73421	0.77690	0.77999	0.87962	0.77479	0.52784
glszm	SmallAreaEmphasis	0.79060	0.10097	0.80504	0.21495	0.45439	0.51503	0.69569	0.73372	0.73889	0.84685	0.75565	0.54720
glszm	SmallAreaHighGrayLevelEmphasis	0.82136	0.09257	0.78335	0.25729	0.55390	0.64290	0.74918	0.76343	0.88790	0.94108	0.87765	0.76779
glszm	SmallAreaLowGrayLevelEmphasis	0.67031	0.32341	0.61222	0.34189	0.57877	0.61149	0.70159	0.65754	0.84272	0.89510	0.84893	0.70177
glszm	ZoneEntropy	0.86861	0.35839	0.87377	0.39969	0.65017	0.74760	0.80559	0.83001	0.85625	0.92591	0.87053	0.74663
glszm	ZonePercentage	0.96277	0.31699	0.95754	0.22228	0.67575	0.78419	0.90772	0.94293	0.92961	0.98386	0.97711	0.93335
glszm	ZoneVariance	0.98802	0.27649	0.98159	0.21573	0.90481	0.72648	0.98170	0.97258	0.86075	0.97733	0.98427	0.94278
ngtdm	Busyness	0.91639	0.40646	0.85422	0.46666	0.61481	0.74905	0.87141	0.86041	0.83308	0.95921	0.92100	0.76593
ngtdm	Coarseness	0.99398	0.14041	0.98757	0.02089	0.79412	0.58168	0.97226	0.97858	0.87055	0.96981	0.97593	0.88172
ngtdm	Complexity	0.96346	0.29310	0.95263	0.15596	0.64000	0.77138	0.90887	0.94454	0.85580	0.96839	0.96970	0.91358
ngtdm	Contrast	0.96238	0.44434	0.96097	0.47685	0.64597	0.76471	0.90104	0.93873	0.96354	0.99247	0.97332	0.90347
ngtdm	Strength	0.98862	0.18235	0.98101	0.15471	0.77487	0.63657	0.98009	0.96690	0.91016	0.98021	0.98878	0.93370

1.00000
0.90000
0.80000
0.70000
0.60000
0.50000
0.40000
0.30000
0.20000
0.10000
0.00000

(B) Heatmap of reproducibility of radiomics features per influencing factor by CCC

Family	Feature	Reposition	High pitch	Tube voltage	Slice thickness	Dose 0.5 mGy	Dose 1.0 mGy	Dose 3.0 mGy	Dose 5.0 mGy	QIR level 0	QIR level 2	Kernel Qr44	Kernel Qr48
firstorder	10Percentile	0.99997	0.21394	0.99991	0.56243	0.99249	0.99673	0.99957	0.99985	0.99980	0.99997	0.99895	0.99525
firstorder	90Percentile	0.99999	0.42398	0.99991	0.48075	0.99507	0.99789	0.99972	0.99992	0.99982	0.99997	0.99953	0.99811
firstorder	Energy	1.00000	0.28502	0.99976	0.26839	0.99880	0.99938	0.99988	0.99996	0.99999	1.00000	0.99982	0.99909
firstorder	Entropy	0.95532	0.54402	0.95955	0.55462	0.76167	0.84946	0.91963	0.93581	0.95067	0.99119	0.97955	0.93283
firstorder	InterquartileRange	0.99978	0.70469	0.99956	0.49903	0.91542	0.96793	0.99635	0.99895	0.99830	0.99973	0.99115	0.96555
firstorder	Kurtosis	0.99759	0.42452	0.99804	0.39154	0.86940	0.95920	0.99399	0.99743	0.94479	0.99285	0.96843	0.86480
firstorder	Maximum	0.99938	0.46210	0.99910	0.48088	0.98781	0.99455	0.99849	0.99893	0.99918	0.99990	0.99675	0.98665
firstorder	MeanAbsoluteDeviation	0.99996	0.65252	0.99975	0.45749	0.92131	0.97001	0.99680	0.99925	0.99832	0.99979	0.99234	0.96915
firstorder	Mean	1.00000	0.31096	0.99996	0.50791	0.99996	0.99996	0.99998	0.99999	1.00000	1.00000	1.00000	1.00000
firstorder	Median	0.99998	0.32654	0.99994	0.48938	0.99976	0.99986	0.99995	0.99997	0.99998	0.99999	0.99994	0.99971
firstorder	Minimum	0.99928	0.15914	0.99901	0.52535	0.97876	0.99019	0.99758	0.99841	0.99905	0.99987	0.99745	0.98978
firstorder	Range	0.99832	0.48079	0.99788	0.35130	0.93020	0.97096	0.99422	0.99649	0.99645	0.99954	0.98937	0.95830
firstorder	RobustMeanAbsoluteDeviation	0.99992	0.69211	0.99973	0.49059	0.91651	0.96812	0.99657	0.99915	0.99851	0.99981	0.99172	0.96652
firstorder	RootMeanSquared	1.00000	0.20430	0.99990	0.26850	0.99619	0.99838	0.99979	0.99994	0.99992	0.99999	0.99946	0.99748
firstorder	Skewness	0.99463	0.60992	0.98796	0.55596	0.88736	0.95411	0.98822	0.99231	0.96243	0.99480	0.99130	0.95683
firstorder	TotalEnergy	1.00000	0.27785	0.99976	0.15381	0.99880	0.99938	0.99988	0.99996	0.99999	1.00000	0.99982	0.99909
firstorder	Uniformity	0.96515	0.57608	0.96641	0.55798	0.79069	0.87889	0.93942	0.94669	0.94154	0.98963	0.97975	0.93067
firstorder	Variance	0.99995	0.70881	0.99968	0.55453	0.86335	0.94339	0.99351	0.99845	0.99899	0.99990	0.98606	0.94215
glcm	Autocorrelation	0.94716	0.45943	0.93294	0.45330	0.76346	0.84192	0.91461	0.92798	0.96988	0.99430	0.97873	0.95041
glcm	ClusterProminence	0.98293	0.82642	0.96876	0.76198	0.82115	0.88930	0.96004	0.96991	0.94698	0.98371	0.98323	0.95181
glcm	ClusterShade	0.98921	0.81682	0.90189	0.49547	0.86614	0.92263	0.97129	0.97760	0.94356	0.98374	0.98675	0.96242
glcm	ClusterTendency	0.97787	0.74816	0.96984	0.69158	0.81439	0.88522	0.95428	0.96412	0.94427	0.98575	0.98237	0.94590
glcm	Contrast	0.96596	0.24024	0.96106	0.15727	0.59465	0.74500	0.90148	0.93795	0.84543	0.96967	0.92157	0.76912
glcm	Correlation	0.99295	0.40335	0.98293	0.25517	0.81791	0.90781	0.97336	0.98752	0.78240	0.94669	0.85499	0.63621
glcm	DifferenceAverage	0.96856	0.30881	0.96234	0.24250	0.66650	0.78959	0.91506	0.94671	0.78509	0.95233	0.93832	0.82721
glcm	DifferenceEntropy	0.96771	0.39537	0.96111	0.23124	0.72961	0.83418	0.92708	0.94864	0.82885	0.96442	0.94128	0.84510
glcm	DifferenceVariance	0.96305	0.31191	0.95832	0.16688	0.60603	0.75822	0.90243	0.93671	0.87638	0.97567	0.91599	0.75532
glcm	Id	0.96906	0.37316	0.95950	0.33838	0.70455	0.80464	0.91693	0.94841	0.71880	0.92836	0.94678	0.86039
glcm	Idm	0.96926	0.35736	0.96082	0.31319	0.69917	0.80443	0.91797	0.94913	0.73372	0.93505	0.94634	0.85770
glcm	Idmn	0.96694	0.25357	0.96231	0.16967	0.61054	0.75682	0.90594	0.94101	0.83737	0.96784	0.92632	0.78546
glcm	Idn	0.96894	0.32733	0.96200	0.26847	0.68086	0.79689	0.91681	0.94798	0.76651	0.94626	0.94175	0.84003
glcm	Imc1	0.99523	0.54659	0.96703	0.12483	0.80070	0.88400	0.96346	0.98449	0.66856	0.91660	0.89353	0.71140
glcm	Imc2	0.99189	0.37629	0.98553	0.29744	0.83161	0.91582	0.97308	0.98570	0.78218	0.94324	0.86327	0.66608
glcm	InverseVariance	0.95627	0.57735	0.93317	0.50469	0.59378	0.70527	0.86478	0.91600	0.58032	0.84377	0.92653	0.81210
glcm	JointAverage	0.94925	0.51429	0.93082	0.50397	0.78694	0.85870	0.92219	0.93028	0.97304	0.99485	0.98132	0.95639
glcm	JointEnergy	0.97333	0.57026	0.97083	0.52237	0.81899	0.90697	0.95940	0.95329	0.89536	0.98048	0.98248	0.94483
glcm	JointEntropy	0.95250	0.45490	0.95404	0.49671	0.74207	0.83322	0.91109	0.93148	0.90507	0.98051	0.97854	0.94032
glcm	MCC	0.99136	0.39765	0.97873	0.24375	0.83737	0.91639	0.97379	0.98556	0.78929	0.94996	0.87517	0.69177
glcm	MaximumProbability	0.94694	0.54860	0.93396	0.55030	0.72395	0.83412	0.90419	0.92584	0.84844	0.96217	0.96443	0.92222
glcm	SumAverage	0.94925	0.51429	0.93082	0.50397	0.78694	0.85870	0.92219	0.93028	0.97304	0.99485	0.98132	0.95639
glcm	SumEntropy	0.96087	0.58036	0.96450	0.55366	0.79505	0.87383	0.93295	0.94380	0.96359	0.99305	0.97505	0.91324
glcm	SumSquares	0.97369	0.69445	0.96879	0.66485	0.78297	0.86407	0.94399	0.95770	0.95726	0.98929	0.98394	0.94795
gldm	DependenceEntropy	0.96801	0.56985	0.96521	0.45184	0.80565	0.88343	0.95019	0.95007	0.86127	0.96978	0.96467	0.88362
gldm	DependenceNonUniformity	0.96278	0.10334	0.94787	0.18039	0.62686	0.73365	0.92945	0.89283	0.68286	0.92337	0.95577	0.88009
gldm	DependenceNonUniformityNormalizec	0.96278	0.10334	0.94787	0.18039	0.62686	0.73365	0.92945	0.89283	0.68286	0.92337	0.95577	0.88009
gldm	DependenceVariance	0.94608	0.37462	0.93276	0.58352	0.48855	0.63766	0.85225	0.90978	0.66778	0.88919	0.95063	0.89614
gldm	GrayLevelNonUniformity	0.96515	0.57608	0.96641	0.55798	0.79069	0.87889	0.93942	0.94669	0.94154	0.98963	0.97975	0.93067
gldm	GrayLevelVariance	0.97355	0.69632	0.96798	0.66673	0.78084	0.86310	0.94377	0.95739	0.95641	0.98908	0.98381	0.94755
gldm	HighGrayLevelEmphasis	0.94976	0.46601	0.93614	0.45288	0.77124	0.84882	0.91855	0.93122	0.97198	0.99467	0.98040	0.95496
gldm	LargeDependenceEmphasis	0.96864	0.46478	0.95447	0.45452	0.72932	0.81260	0.91598	0.94620	0.67691	0.90552	0.95277	0.88773
gldm	LargeDependenceHighGrayLevelEmpha	0.93700	0.49620	0.94092	0.59262	0.51902	0.66969	0.86577	0.92343	0.70450	0.91677	0.94525	0.87292
gldm	LargeDependenceLowGrayLevelEmphas	0.98060	0.64354	0.97240	0.51816	0.84605	0.94679	0.98476	0.97197	0.92696	0.98859	0.99412	0.96916
gldm	LowGrayLevelEmphasis	0.96515	0.71913	0.95338	0.63371	0.84991	0.92398	0.96147	0.95442	0.96737	0.99391	0.98853	0.95778
gldm	SmallDependenceEmphasis	0.96030	0.23616	0.95387	0.23751	0.61120	0.73505	0.89125	0.93337	0.70392	0.92627	0.90723	0.73680
gldm	SmallDependenceHighGrayLevelEmpha	0.95631	0.30411	0.94517	0.24350	0.65926	0.78171	0.90515	0.92948	0.83123	0.95905	0.94113	0.82691
gldm	SmallDependenceLowGrayLevelEmphas	0.68993	0.08631	0.64136	0.24828	0.31581	0.42014	0.58811	0.59940	0.63040	0.87667	0.80014	0.52546
glrlm	GrayLevelNonUniformity	0.97382	0.59018	0.95379	0.36003	0.76693	0.85755	0.93597	0.95102	0.71148	0.91549	0.92156	0.77117
glrlm	GrayLevelNonUniformityNormalized	0.96075	0.54218	0.96152	0.55881	0.78348	0.85790	0.92820	0.94189	0.95378	0.99222	0.98019	0.93346
glrlm	GrayLevelVariance	0.95982	0.64382	0.94906	0.59777	0.72896	0.82680	0.91987	0.93619	0.94024	0.98465	0.97642	0.92624
glrlm	HighGrayLevelRunEmphasis	0.93886	0.41475	0.92105	0.44864	0.75013	0.83112	0.90245	0.91581	0.97140	0.99431	0.97588	0.94465
glrlm	LongRunEmphasis	0.99138	0.11125	0.98235	0.09458	0.84357	0.73037	0.98828	0.95415	0.91844	0.98396	0.97437	0.89491
glrlm	LongRunHighGrayLevelEmphasis	0.91150	0.39631	0.91642	0.42445	0.40399	0.52474	0.81183	0.88612	0.56086	0.83927	0.90805	0.79990
glrlm	LongRunLowGrayLevelEmphasis	0.99193	0.08081	0.98302	0.04630	0.84019	0.73053	0.98957	0.95450	0.92245	0.98552	0.97576	0.89700
glrlm	LowGrayLevelRunEmphasis	0.96421	0.74339	0.94989	0.67701	0.86286	0.91897	0.95546	0.94838	0.96827	0.99399	0.98613	0.96136
glrlm	RunEntropy	0.98794	0.54761	0.95174	0.17509	0.73605	0.82171	0.93530	0.96568	0.53472	0.85428	0.89166	0.68812
glrlm	RunLengthNonUniformity	0.96455	0.31011	0.95540	0.33622	0.65719	0.76362	0.89900	0.94105	0.65829	0.90902	0.93838	0.82926
glrlm	RunLengthNonUniformityNormalized	0.96518	0.37219	0.95501	0.36209	0.68628	0.78038	0.90363	0.94285	0.65450	0.90524	0.93562	0.82340
glrlm	RunPercentage	0.96835	0.41774	0.95571	0.40771	0.71267	0.80150	0.91300	0.94627	0.67470	0.90879	0.94841	0.86974
glrlm	RunVariance	0.97967	0.23672	0.95627	0.17735	0.78092	0.86242	0.98202	0.86603	0.83809	0.96561	0.96753	0.89291
glrlm	ShortRunEmphasis	0.96540	0.43015	0.95383	0.38883	0.72046	0.79761	0.90951	0.94451	0.65360	0.90067	0.93210	0.82313
glrlm	ShortRunHighGrayLevelEmphasis	0.94693	0.38334	0.92491	0.37842	0.76344	0.84095	0.90842	0.92067	0.94179	0.98487	0.97374	0.93552
glrlm	ShortRunLowGrayLevelEmphasis	0.85376	0.41304	0.81930	0.48238	0.65753	0.75217	0.84044	0.81882	0.91913	0.97631	0.94407	0.84545
glszm	GrayLevelNonUniformity	0.96841	0.25013	0.96081	0.15726	0.68365	0.79710	0.92575	0.95358	0.70590	0.92765	0.90931	0.74768
glszm	GrayLevelNonUniformityNormalized	0.90779	0.64277	0.93138	0.50097	0.71214	0.84553	0.90776	0.92681	0.92548	0.97849	0.93899	0.85313
glszm	GrayLevelVariance	0.78446	0.52019	0.75686	0.24526	0.41507	0.57516	0.69606	0.71653	0.76983	0.89952	0.87167	0.75847
glszm	HighGrayLevelZoneEmphasis	0.89002	0.34352	0.85203	0.44237	0.68473	0.75619	0.83624	0.84562	0.95567	0.98628	0.95533	0.90413
glszm	LargeAreaEmphasis	0.98899	0.25739	0.98285	0.20015	0.90289	0.69593	0.98133	0.97479	0.84636	0.97559	0.98353	0.93774
glszm	LargeAreaHighGrayLevelEmphasis	0.82754	0.07614	0.81014	0.23634	0.33025	0.53494	0.73917	0.82078	0.45340	0.79224	0.84427	0.75301
glszm	LargeAreaLowGrayLevelEmphasis	0.98918	0.16182	0.98234	0.10173	0.88365	0.67136	0.98564	0.97677	0.84755	0.97798	0.98502	0.93557
glszm	LowGrayLevelZoneEmphasis	0.86259	0.46467	0.81295	0.48317	0.73681	0.79775	0.86445	0.84097	0.92684	0.98270	0.94412	0.82289
glszm	SizeZoneNonUniformity	0.94095	0.08096	0.93743	0.14504	0.47332	0.61284	0.83632	0.89584	0.71000	0.92250	0.84161	0.58170
glszm	SizeZoneNonUniformityNormalized	0.83935	0.03951	0.85529	0.17843	0.37093	0.49566	0.73008	0.77445	0.66061	0.84323	0.63885	0.27413
glszm	SmallAreaEmphasis	0.78995	0.08168	0.80487	0.21469	0.38769	0.49355	0.69323	0.73147	0.61907	0.81004	0.61344	0.27860
glszm	SmallAreaHighGrayLevelEmphasis	0.82096	0.08353	0.78214	0.25704	0.51124	0.63400	0.74725	0.76292	0.85004	0.93081	0.83672	0.65002
glszm	SmallAreaLowGrayLevelEmphasis	0.66947	0.31306	0.60561	0.34026	0.57722	0.61090	0.70148	0.65696	0.82018	0.88796	0.82225	0.62512
glszm	ZoneEntropy	0.86843	0.33535	0.87349	0.39647	0.55995	0.70645	0.79652	0.82894	0.83861	0.92168	0.81505	0.56565
glszm	ZonePercentage	0.96261	0.28705	0.95718	0.22095	0.65043	0.77744	0.90758	0.94187	0.75172	0.94383	0.93306	0.81396
glszm	ZoneVariance	0.98799	0.27633	0.98149	0.21488	0.90288	0.72500	0.98163	0.97253	0.85458	0.97578	0.98417	0.94256
ngtdm	Busyness	0.91638	0.36684	0.85369	0.46337	0.58923	0.74236	0.87027	0.85965	0.78513	0.94358	0.86368	0.63659
ngtdm	Coarseness	0.99397	0.13336	0.98733	0.02072	0.79275	0.58154	0.97223	0.97826	0.83607	0.96015	0.95270	0.82995
ngtdm	Complexity	0.96336	0.23019	0.95205	0.15404	0.59252	0.75511	0.90717	0.94381	0.69873	0.92635	0.85244	0.63715
ngtdm	Contrast	0.96226	0.41738	0.96095	0.47304	0.58903	0.73547	0.89597	0.93399	0.94826	0.98978	0.95302	0.84405
ngtdm	Strength	0.98862	0.18112	0.98083	0.15394	0.77382	0.63490	0.97994	0.96674	0.89649	0.97692	0.98547	0.92731

1.00000
0.90000
0.80000
0.70000
0.60000
0.50000
0.40000
0.30000
0.20000
0.10000
0.00000

Supplementary Figure S2 Heatmap of variability of radiomics features per influencing factor

(A) Heatmap of reproducibility of radiomics features per influencing factor by CV

Family	Feature	Reposition	High pitch	Tube voltage	Slice thickness	Dose 0.5 mGy	Dose 1.0 mGy	Dose 3.0 mGy	Dose 5.0 mGy	QIR level 0	QIR level 2	Kernel Qr44	Kernel Qr48
firstorder	10Percentile	11%	75%	10%	39%	21%	81%	13%	11%	10%	9%	9%	11%
firstorder	90Percentile	7%	50%	7%	60%	21%	14%	8%	7%	8%	7%	8%	13%
firstorder	Energy	7%	47%	8%	39%	12%	11%	9%	8%	9%	8%	8%	11%
firstorder	Entropy	6%	9%	6%	8%	7%	7%	6%	6%	6%	6%	6%	7%
firstorder	InterquartileRange	6%	36%	8%	38%	18%	14%	9%	8%	12%	8%	9%	13%
firstorder	Kurtosis	17%	30%	16%	24%	22%	20%	18%	17%	17%	16%	17%	19%
firstorder	Maximum	20%	86%	20%	240%	98%	105%	70%	24%	17%	18%	17%	20%
firstorder	MeanAbsoluteDeviation	5%	35%	7%	37%	17%	13%	9%	7%	11%	7%	9%	13%
firstorder	Mean	6%	57%	6%	34%	6%	6%	6%	6%	6%	6%	6%	6%
firstorder	Median	9%	59%	9%	38%	9%	9%	9%	9%	9%	9%	10%	13%
firstorder	Minimum	7%	207%	7%	450%	11%	10%	8%	7%	7%	7%	7%	9%
firstorder	Range	11%	36%	12%	38%	21%	18%	14%	13%	15%	13%	14%	18%
firstorder	RobustMeanAbsoluteDeviation	6%	35%	7%	37%	17%	14%	9%	8%	12%	8%	9%	13%
firstorder	RootMeanSquared	4%	33%	4%	24%	6%	5%	4%	4%	4%	4%	4%	5%
firstorder	Skewness	66%	169%	57%	223%	103%	73%	67%	60%	63%	55%	68%	653%
firstorder	TotalEnergy	7%	48%	8%	53%	12%	11%	9%	8%	9%	8%	8%	11%
firstorder	Uniformity	9%	15%	9%	14%	12%	11%	10%	10%	9%	9%	9%	10%
firstorder	Variance	12%	54%	15%	55%	32%	27%	18%	16%	22%	15%	18%	26%
glcm	Autocorrelation	15%	21%	15%	20%	18%	16%	15%	15%	14%	14%	15%	15%
glcm	ClusterProminence	27%	37%	27%	40%	32%	30%	28%	28%	29%	28%	30%	35%
glcm	ClusterShade	64%	100%	66%	207%	185%	101%	98%	80%	67%	65%	57%	61%
glcm	ClusterTendency	17%	25%	17%	25%	21%	19%	18%	18%	17%	17%	18%	21%
glcm	Contrast	15%	28%	15%	28%	22%	20%	17%	16%	20%	17%	18%	22%
glcm	Correlation	5%	12%	5%	13%	8%	7%	5%	5%	11%	7%	11%	23%
glcm	DifferenceAverage	10%	19%	10%	18%	14%	13%	11%	10%	13%	11%	11%	14%
glcm	DifferenceEntropy	6%	11%	6%	10%	8%	7%	6%	6%	7%	6%	7%	8%
glcm	DifferenceVariance	13%	23%	13%	23%	18%	16%	14%	13%	15%	13%	15%	19%
glcm	Id	3%	6%	3%	6%	5%	4%	4%	3%	5%	4%	4%	5%
glcm	Idm	4%	8%	4%	8%	6%	5%	4%	4%	6%	5%	5%	6%
glcm	Idmn	0%	1%	0%	1%	0%	0%	0%	0%	0%	0%	0%	1%
glcm	Idn	1%	2%	1%	2%	1%	1%	1%	1%	1%	1%	1%	1%
glcm	Imc1	9%	24%	9%	27%	16%	14%	11%	9%	22%	13%	19%	32%
glcm	Imc2	4%	10%	4%	11%	7%	6%	5%	4%	9%	6%	8%	15%
glcm	InverseVariance	5%	7%	5%	6%	7%	6%	6%	5%	6%	6%	5%	7%
glcm	JointAverage	8%	11%	8%	11%	9%	9%	8%	8%	7%	8%	8%	8%
glcm	JointEnergy	18%	30%	18%	27%	24%	22%	20%	19%	20%	18%	19%	20%
glcm	JointEntropy	6%	9%	6%	8%	7%	7%	7%	6%	6%	6%	6%	7%
glcm	MCC	5%	11%	5%	13%	7%	6%	5%	5%	10%	6%	10%	17%
glcm	MaximumProbability	18%	28%	19%	26%	24%	22%	20%	19%	22%	19%	19%	21%
glcm	SumAverage	8%	11%	8%	11%	9%	9%	8%	8%	7%	8%	8%	8%
glcm	SumEntropy	5%	7%	5%	7%	6%	5%	5%	5%	5%	5%	5%	6%
glcm	SumSquares	16%	24%	16%	24%	21%	19%	17%	17%	16%	16%	17%	18%
gldm	DependenceEntropy	3%	4%	3%	4%	3%	3%	3%	3%	3%	3%	3%	4%
gldm	DependenceNonUniformity	7%	11%	7%	10%	9%	9%	8%	7%	11%	8%	8%	9%
gldm	DependenceNonUniformityNormalizec	7%	11%	7%	10%	9%	9%	8%	7%	11%	8%	8%	9%
gldm	DependenceVariance	9%	16%	10%	14%	14%	13%	11%	10%	16%	12%	11%	12%
gldm	GrayLevelNonUniformity	9%	15%	9%	14%	12%	11%	10%	10%	9%	9%	9%	10%
gldm	GrayLevelVariance	16%	23%	16%	24%	21%	19%	17%	17%	16%	16%	17%	18%
gldm	HighGrayLevelEmphasis	14%	20%	15%	20%	17%	16%	15%	15%	14%	14%	14%	15%
gldm	LargeDependenceEmphasis	11%	21%	11%	19%	17%	15%	13%	11%	19%	14%	13%	15%
gldm	LargeDependenceHighGrayLevelEmpha	17%	26%	18%	23%	24%	23%	19%	18%	23%	19%	19%	21%
gldm	LargeDependenceLowGrayLevelEmpha	28%	47%	29%	46%	34%	32%	29%	30%	35%	30%	30%	32%
gldm	LowGrayLevelEmphasis	23%	31%	23%	30%	25%	25%	23%	24%	22%	22%	23%	24%
gldm	SmallDependenceEmphasis	12%	23%	12%	21%	17%	16%	13%	12%	18%	14%	14%	18%
gldm	SmallDependenceHighGrayLevelEmpha	18%	30%	18%	29%	24%	21%	19%	19%	23%	19%	19%	22%
gldm	SmallDependenceLowGrayLevelEmpha	24%	31%	24%	27%	29%	28%	26%	25%	27%	25%	25%	30%
glrlm	GrayLevelNonUniformity	5%	8%	5%	9%	7%	6%	5%	5%	8%	6%	6%	8%
glrlm	GrayLevelNonUniformityNormalized	8%	12%	7%	11%	10%	9%	8%	8%	8%	8%	8%	8%
glrlm	GrayLevelVariance	13%	18%	13%	19%	17%	15%	14%	14%	13%	13%	14%	15%
glrlm	HighGrayLevelRunEmphasis	13%	19%	14%	18%	16%	15%	14%	14%	13%	13%	13%	13%
glrlm	LongRunEmphasis	20%	34%	21%	31%	27%	26%	23%	21%	29%	23%	22%	25%
glrlm	LongRunHighGrayLevelEmphasis	21%	30%	21%	27%	27%	26%	23%	22%	27%	23%	22%	25%
glrlm	LongRunLowGrayLevelEmphasis	35%	58%	37%	54%	42%	40%	38%	38%	43%	38%	37%	40%
glrlm	LowGrayLevelRunEmphasis	22%	29%	22%	28%	24%	24%	22%	23%	21%	22%	22%	23%
glrlm	RunEntropy	1%	3%	2%	4%	2%	2%	2%	1%	3%	2%	2%	3%
glrlm	RunLengthNonUniformity	12%	22%	13%	20%	17%	16%	14%	13%	18%	14%	14%	16%
glrlm	RunLengthNonUniformityNormalized	7%	13%	7%	12%	10%	9%	8%	8%	11%	8%	8%	10%
glrlm	RunPercentage	6%	11%	6%	9%	8%	7%	7%	6%	8%	7%	7%	8%
glrlm	RunVariance	26%	46%	27%	41%	36%	34%	31%	26%	38%	29%	28%	34%
glrlm	ShortRunEmphasis	4%	8%	4%	7%	6%	5%	5%	4%	6%	5%	5%	6%
glrlm	ShortRunHighGrayLevelEmphasis	14%	21%	14%	20%	17%	15%	14%	14%	14%	14%	14%	14%
glrlm	ShortRunLowGrayLevelEmphasis	21%	26%	22%	25%	24%	23%	22%	22%	21%	21%	21%	23%
glszm	GrayLevelNonUniformity	11%	24%	11%	22%	17%	15%	12%	11%	18%	13%	13%	17%
glszm	GrayLevelNonUniformityNormalized	8%	9%	8%	9%	8%	8%	8%	8%	8%	8%	9%	9%
glszm	GrayLevelVariance	10%	10%	10%	11%	10%	10%	10%	10%	9%	9%	10%	10%
glszm	HighGrayLevelZoneEmphasis	12%	15%	12%	14%	13%	12%	12%	12%	11%	11%	11%	12%
glszm	LargeAreaEmphasis	55%	96%	59%	89%	85%	74%	63%	60%	72%	61%	64%	70%
glszm	LargeAreaHighGrayLevelEmphasis	54%	91%	56%	82%	81%	72%	61%	58%	69%	58%	62%	68%
glszm	LargeAreaLowGrayLevelEmphasis	63%	120%	70%	102%	92%	82%	71%	69%	81%	70%	73%	80%
glszm	LowGrayLevelZoneEmphasis	23%	29%	23%	28%	25%	24%	23%	24%	22%	22%	23%	24%
glszm	SizeZoneNonUniformity	21%	38%	21%	34%	30%	27%	23%	22%	29%	23%	25%	32%
glszm	SizeZoneNonUniformityNormalized	11%	18%	11%	14%	14%	13%	12%	12%	12%	11%	13%	18%
glszm	SmallAreaEmphasis	7%	12%	7%	8%	9%	8%	8%	8%	8%	7%	8%	11%
glszm	SmallAreaHighGrayLevelEmphasis	15%	20%	14%	17%	17%	16%	15%	15%	14%	14%	15%	16%
glszm	SmallAreaLowGrayLevelEmphasis	26%	31%	26%	29%	29%	28%	27%	28%	25%	26%	27%	29%
glszm	ZoneEntropy	3%	4%	3%	3%	3%	3%	3%	3%	3%	3%	3%	5%
glszm	ZonePercentage	15%	27%	15%	26%	21%	19%	16%	15%	21%	16%	16%	19%
glszm	ZoneVariance	60%	100%	65%	94%	91%	80%	68%	65%	77%	66%	70%	76%
ngtdm	Busyness	11%	17%	11%	14%	14%	14%	12%	11%	13%	11%	13%	16%
ngtdm	Coarseness	9%	15%	9%	14%	12%	11%	9%	10%	12%	10%	12%	17%
ngtdm	Complexity	7%	15%	7%	14%	11%	10%	8%	7%	10%	8%	10%	15%
ngtdm	Contrast	22%	33%	22%	32%	29%	26%	24%	23%	23%	23%	24%	26%
ngtdm	Strength	14%	22%	14%	19%	17%	17%	15%	15%	18%	15%	16%	20%

20%
15%
10%
5%
0%

(B) Heatmap of reproducibility of radiomics features per influencing factor by QCD

Family	Feature	Reposition	High pitch	Tube voltage	Slice thickness	Dose 0.5 mGy	Dose 1.0 mGy	Dose 3.0 mGy	Dose 5.0 mGy	QIR level 0	QIR level 2	Kernel Qr44	Kernel Qr48
firstorder	10Percentile	8%	70%	6%	37%	17%	29%	10%	8%	8%	7%	6%	9%
firstorder	90Percentile	5%	48%	5%	59%	19%	12%	6%	5%	5%	5%	6%	11%
firstorder	Energy	5%	45%	5%	37%	10%	8%	6%	5%	6%	5%	5%	8%
firstorder	Entropy	4%	6%	4%	6%	5%	4%	4%	4%	4%	4%	4%	4%
firstorder	InterquartileRange	4%	34%	5%	36%	16%	12%	7%	6%	10%	6%	7%	12%
firstorder	Kurtosis	9%	15%	9%	15%	12%	11%	9%	9%	9%	9%	9%	10%
firstorder	Maximum	13%	73%	13%	326%	70%	217%	110%	15%	11%	13%	12%	15%
firstorder	MeanAbsoluteDeviation	4%	33%	5%	36%	15%	12%	6%	5%	10%	6%	7%	11%
firstorder	Mean	4%	55%	4%	33%	4%	4%	4%	4%	4%	4%	4%	4%
firstorder	Median	7%	57%	7%	36%	6%	6%	7%	7%	7%	7%	8%	9%
firstorder	Minimum	4%	260%	4%	413%	9%	7%	5%	5%	5%	4%	5%	7%
firstorder	Range	7%	32%	8%	35%	16%	13%	10%	9%	11%	8%	9%	13%
firstorder	RobustMeanAbsoluteDeviation	4%	34%	5%	36%	15%	12%	7%	5%	10%	6%	7%	11%
firstorder	RootMeanSquared	2%	32%	2%	23%	5%	4%	3%	3%	3%	3%	3%	4%
firstorder	Skewness	102%	109%	38%	128%	69%	45%	41%	36%	43%	34%	147%	72%
firstorder	TotalEnergy	5%	47%	5%	50%	10%	8%	6%	5%	6%	5%	5%	8%
firstorder	Uniformity	6%	11%	5%	10%	8%	7%	6%	6%	6%	6%	6%	7%
firstorder	Variance	9%	50%	10%	51%	28%	23%	13%	11%	18%	12%	14%	22%
glcm	Autocorrelation	9%	16%	10%	16%	11%	10%	10%	10%	9%	9%	10%	10%
glcm	ClusterProminence	17%	28%	18%	32%	23%	21%	18%	19%	19%	17%	19%	22%
glcm	ClusterShade	40%	75%	45%	107%	272%	70%	75%	53%	42%	42%	34%	37%
glcm	ClusterTendency	11%	19%	11%	20%	15%	14%	12%	12%	12%	11%	11%	14%
glcm	Contrast	10%	24%	10%	24%	17%	14%	12%	11%	14%	11%	13%	17%
glcm	Correlation	3%	10%	3%	12%	7%	5%	4%	3%	10%	5%	9%	21%
glcm	DifferenceAverage	6%	15%	7%	15%	11%	9%	8%	7%	10%	8%	8%	11%
glcm	DifferenceEntropy	4%	9%	4%	8%	6%	5%	4%	4%	5%	4%	4%	6%
glcm	DifferenceVariance	8%	19%	8%	20%	14%	11%	9%	9%	11%	9%	10%	15%
glcm	Id	2%	5%	2%	5%	4%	3%	2%	2%	4%	3%	3%	4%
glcm	Idm	2%	6%	2%	6%	4%	4%	3%	3%	5%	3%	3%	5%
glcm	Idmn	0%	0%	0%	1%	0%	0%	0%	0%	0%	0%	0%	0%
glcm	Idn	0%	1%	0%	1%	1%	1%	1%	1%	1%	1%	1%	1%
glcm	Imc1	6%	22%	6%	24%	14%	11%	7%	6%	19%	10%	16%	30%
glcm	Imc2	3%	9%	3%	10%	5%	4%	3%	3%	8%	4%	7%	14%
glcm	InverseVariance	3%	5%	3%	4%	5%	5%	4%	3%	5%	4%	4%	5%
glcm	JointAverage	5%	8%	5%	9%	6%	6%	5%	5%	5%	5%	5%	5%
glcm	JointEnergy	11%	21%	11%	20%	16%	14%	12%	12%	13%	12%	12%	13%
glcm	JointEntropy	4%	7%	4%	6%	5%	5%	4%	4%	4%	4%	4%	5%
glcm	MCC	3%	10%	3%	12%	6%	5%	4%	3%	9%	5%	8%	16%
glcm	MaximumProbability	12%	20%	12%	19%	17%	15%	13%	12%	14%	13%	12%	14%
glcm	SumAverage	5%	8%	5%	9%	6%	6%	5%	5%	5%	5%	5%	5%
glcm	SumEntropy	3%	5%	3%	5%	4%	4%	3%	3%	3%	3%	3%	4%
glcm	SumSquares	10%	18%	10%	19%	15%	13%	11%	11%	11%	11%	11%	12%
gldm	DependenceEntropy	2%	3%	2%	3%	2%	2%	2%	2%	2%	2%	2%	2%
gldm	DependenceNonUniformity	4%	7%	4%	6%	6%	5%	4%	4%	7%	5%	4%	5%
gldm	DependenceNonUniformityNormalizec	4%	7%	4%	6%	6%	5%	4%	4%	7%	5%	4%	5%
gldm	DependenceVariance	6%	12%	7%	11%	11%	10%	8%	7%	13%	8%	7%	8%
gldm	GrayLevelNonUniformity	6%	11%	5%	10%	8%	7%	6%	6%	6%	6%	6%	7%
gldm	GrayLevelVariance	10%	18%	10%	19%	15%	13%	11%	11%	11%	11%	11%	12%
gldm	HighGrayLevelEmphasis	9%	16%	10%	16%	11%	10%	9%	9%	9%	9%	9%	10%
gldm	LargeDependenceEmphasis	7%	17%	7%	16%	12%	11%	9%	8%	15%	10%	9%	11%
gldm	LargeDependenceHighGrayLevelEmpha	11%	18%	12%	17%	16%	15%	13%	12%	17%	13%	13%	15%
gldm	LargeDependenceLowGrayLevelEmpha	17%	29%	17%	32%	21%	19%	17%	17%	22%	17%	17%	19%
gldm	LowGrayLevelEmphasis	15%	22%	15%	22%	17%	17%	15%	15%	15%	15%	15%	15%
gldm	SmallDependenceEmphasis	8%	19%	8%	18%	13%	11%	9%	8%	14%	10%	10%	14%
gldm	SmallDependenceHighGrayLevelEmpha	13%	25%	13%	24%	18%	15%	13%	13%	17%	13%	13%	16%
gldm	SmallDependenceLowGrayLevelEmpha	16%	22%	16%	20%	20%	19%	18%	17%	19%	17%	18%	21%
glrlm	GrayLevelNonUniformity	3%	6%	3%	8%	5%	4%	4%	3%	6%	4%	4%	6%
glrlm	GrayLevelNonUniformityNormalized	5%	8%	5%	8%	7%	6%	5%	5%	5%	5%	5%	5%
glrlm	GrayLevelVariance	8%	13%	9%	15%	12%	10%	9%	9%	9%	9%	9%	9%
glrlm	HighGrayLevelRunEmphasis	9%	14%	9%	14%	10%	10%	9%	9%	8%	8%	8%	9%
glrlm	LongRunEmphasis	9%	21%	10%	19%	15%	14%	11%	10%	17%	12%	11%	14%
glrlm	LongRunHighGrayLevelEmphasis	11%	18%	12%	16%	16%	15%	13%	12%	17%	13%	13%	15%
glrlm	LongRunLowGrayLevelEmphasis	18%	31%	18%	33%	21%	20%	18%	18%	22%	18%	18%	20%
glrlm	LowGrayLevelRunEmphasis	14%	20%	15%	20%	17%	16%	14%	15%	14%	14%	14%	15%
glrlm	RunEntropy	1%	3%	1%	3%	2%	2%	1%	1%	3%	2%	2%	3%
glrlm	RunLengthNonUniformity	8%	18%	9%	17%	13%	12%	10%	9%	15%	10%	10%	12%
glrlm	RunLengthNonUniformityNormalized	5%	11%	5%	10%	8%	7%	5%	5%	9%	6%	6%	7%
glrlm	RunPercentage	4%	8%	4%	7%	6%	5%	5%	4%	7%	5%	4%	6%
glrlm	RunVariance	14%	30%	14%	28%	22%	20%	16%	15%	24%	18%	16%	20%
glrlm	ShortRunEmphasis	3%	6%	3%	6%	4%	4%	3%	3%	5%	3%	3%	4%
glrlm	ShortRunHighGrayLevelEmphasis	9%	16%	9%	16%	11%	10%	9%	9%	9%	9%	9%	9%
glrlm	ShortRunLowGrayLevelEmphasis	14%	18%	14%	18%	16%	16%	15%	14%	13%	14%	14%	15%
glszm	GrayLevelNonUniformity	7%	20%	7%	19%	13%	11%	8%	7%	15%	8%	9%	14%
glszm	GrayLevelNonUniformityNormalized	5%	6%	5%	5%	5%	5%	5%	5%	5%	5%	5%	6%
glszm	GrayLevelVariance	6%	6%	6%	7%	6%	6%	6%	6%	6%	6%	6%	7%
glszm	HighGrayLevelZoneEmphasis	8%	11%	8%	10%	8%	8%	8%	8%	7%	7%	7%	7%
glszm	LargeAreaEmphasis	30%	53%	31%	54%	45%	40%	35%	32%	43%	34%	32%	37%
glszm	LargeAreaHighGrayLevelEmphasis	29%	50%	30%	50%	45%	38%	34%	31%	41%	34%	32%	36%
glszm	LargeAreaLowGrayLevelEmphasis	32%	56%	33%	54%	45%	42%	35%	33%	44%	35%	34%	37%
glszm	LowGrayLevelZoneEmphasis	14%	18%	15%	19%	16%	15%	15%	15%	14%	14%	14%	15%
glszm	SizeZoneNonUniformity	14%	32%	14%	28%	23%	20%	16%	15%	23%	16%	18%	26%
glszm	SizeZoneNonUniformityNormalized	7%	13%	7%	10%	10%	9%	7%	7%	8%	7%	9%	14%
glszm	SmallAreaEmphasis	5%	9%	5%	6%	6%	5%	5%	5%	5%	4%	6%	9%
glszm	SmallAreaHighGrayLevelEmphasis	10%	15%	10%	13%	11%	10%	10%	10%	9%	10%	9%	11%
glszm	SmallAreaLowGrayLevelEmphasis	17%	19%	16%	19%	19%	18%	18%	17%	16%	16%	17%	19%
glszm	ZoneEntropy	2%	3%	2%	2%	2%	2%	2%	2%	2%	2%	2%	3%
glszm	ZonePercentage	10%	23%	10%	22%	16%	14%	11%	10%	17%	11%	11%	15%
glszm	ZoneVariance	33%	56%	34%	57%	49%	43%	38%	35%	46%	37%	35%	40%
ngtdm	Busyness	7%	13%	7%	10%	11%	9%	8%	7%	9%	7%	8%	12%
ngtdm	Coarseness	3%	9%	3%	9%	6%	5%	3%	3%	6%	4%	6%	12%
ngtdm	Complexity	4%	12%	5%	11%	9%	7%	5%	5%	8%	6%	7%	12%
ngtdm	Contrast	14%	26%	14%	26%	22%	19%	15%	15%	15%	15%	15%	18%
ngtdm	Strength	7%	14%	7%	11%	9%	8%	7%	6%	10%	7%	9%	13%

20%
15%
10%
5%
0%

Supplementary Figure S3 Heatmap of variability of radiomics features according to materials

(A) Heatmap of reproducibility of radiomics features according to materials by CV

Family	Feature	ROI_1	ROI_2	ROI_3	ROI_4	ROI_5	ROI_6	ROI_7	ROI_8	ROI_9	ROI_10	ROI_11	ROI_12	ROI_13	ROI_14	ROI_15	ROI_16	ROI_17	ROI_18	ROI_19	ROI_20	ROI_21	ROI_22	ROI_23	ROI_24	ROI_25	ROI_26	ROI_27	ROI_28
firstorder	10Percentile	6%	2%	14%	98%	12%	133%	69%	12%	2%	14%	12%	11%	7%	25%	34%	171%	40%	28%	27%	96%	22%	2%	0%	20%	270%	29%	22%	83%
firstorder	90Percentile	7%	2%	70%	134%	32%	119%	36%	126%	44%	24%	17%	10%	6%	69%	276%	30%	14%	278%	48%	168%	28%	2%	0%	89%	222%	28%	31%	130%
firstorder	Energy	13%	1%	23%	149%	20%	207%	39%	60%	19%	30%	22%	17%	10%	83%	303%	32%	46%	159%	40%	211%	28%	3%	0%	67%	333%	29%	15%	56%
firstorder	Entropy	2%	6%	3%	8%	3%	7%	12%	2%	3%	7%	5%	4%	6%	9%	13%	5%	42%	2%	7%	9%	5%	9%	5%	5%	7%	10%	14%	8%
firstorder	InterquartileRange	10%	15%	20%	32%	17%	32%	104%	28%	128%	66%	16%	16%	25%	29%	119%	34%	14%	29%	245%	45%	48%	12%	27%	20%	32%	25%	116%	34%
firstorder	Kurtosis	7%	17%	9%	28%	8%	19%	30%	8%	8%	12%	13%	9%	26%	30%	32%	11%	133%	7%	27%	25%	18%	36%	19%	12%	69%	25%	82%	21%
firstorder	Maximum	7%	5%	24%	64%	15%	70%	30%	82%	55%	51%	27%	10%	14%	396%	123%	28%	67%	147%	54%	282%	32%	11%	1%	210%	221%	28%	111%	226%
firstorder	MeanAbsoluteDeviation	7%	15%	18%	30%	15%	29%	98%	27%	130%	67%	16%	14%	18%	29%	117%	32%	15%	29%	235%	46%	54%	12%	26%	19%	32%	25%	113%	32%
firstorder	Mean	12%	1%	18%	362%	10%	27%	50%	90%	23%	15%	14%	10%	5%	39%	33%	32%	21%	144%	37%	126%	25%	2%	0%	34%	584%	29%	2%	100%
firstorder	Median	30%	1%	22%	539%	15%	24%	48%	152%	27%	16%	15%	10%	5%	41%	39%	31%	28%	224%	39%	130%	26%	2%	0%	37%	580%	29%	2%	102%
firstorder	Minimum	3%	8%	11%	62%	8%	66%	108%	10%	12%	21%	9%	11%	7%	17%	57%	32%	25%	9%	18%	51%	15%	2%	1%	10%	158%	30%	37%	70%
firstorder	Range	3%	21%	12%	24%	9%	21%	69%	25%	118%	67%	20%	16%	9%	32%	86%	28%	60%	27%	190%	49%	80%	18%	27%	18%	42%	34%	90%	27%
firstorder	RobustMeanAbsoluteDeviation	9%	15%	19%	32%	16%	31%	103%	28%	127%	65%	16%	15%	23%	29%	118%	33%	14%	29%	242%	45%	48%	12%	26%	20%	31%	25%	115%	33%
firstorder	RootMeanSquared	6%	1%	12%	53%	10%	77%	30%	24%	12%	13%	13%	10%	6%	35%	137%	27%	21%	56%	32%	120%	25%	2%	0%	28%	197%	28%	7%	26%
firstorder	Skewness	22%	32%	25%	57%	20%	62%	236%	24%	87%	265%	106%	90%	134%	62%	349%	31%	89%	48%	200%	59%	107%	43%	110%	40%	566%	99%	114%	86%
firstorder	TotalEnergy	21%	17%	25%	139%	29%	128%	41%	23%	25%	29%	31%	26%	23%	65%	298%	35%	47%	81%	41%	213%	29%	18%	17%	45%	331%	34%	11%	48%
firstorder	Uniformity	9%	11%	8%	21%	8%	16%	20%	5%	7%	14%	9%	10%	17%	17%	20%	11%	38%	6%	14%	15%	11%	15%	11%	11%	12%	17%	26%	18%
firstorder	Variance	12%	29%	35%	44%	26%	49%	194%	35%	298%	173%	28%	23%	29%	58%	279%	43%	56%	36%	337%	75%	160%	25%	55%	36%	65%	42%	213%	45%
glcm	Autocorrelation	5%	14%	7%	15%	6%	16%	37%	10%	15%	17%	28%	14%	14%	20%	29%	11%	76%	13%	25%	20%	19%	27%	18%	11%	20%	24%	22%	18%
glcm	ClusterProminence	15%	34%	27%	38%	24%	44%	78%	20%	30%	52%	34%	28%	25%	65%	70%	24%	53%	21%	110%	41%	40%	35%	31%	45%	35%	49%	56%	41%
glcm	ClusterShade	24%	43%	36%	45%	29%	45%	525%	28%	82%	184%	80%	332%	157%	57%	181%	34%	46%	53%	242%	66%	69%	47%	138%	40%	590%	147%	78%	73%
glcm	ClusterTendency	10%	20%	15%	24%	15%	26%	43%	11%	16%	29%	19%	16%	18%	39%	40%	14%	63%	11%	47%	26%	25%	23%	17%	25%	20%	30%	35%	27%
glcm	Contrast	15%	20%	18%	27%	19%	25%	35%	27%	56%	29%	27%	21%	38%	32%	34%	29%	51%	26%	24%	28%	34%	23%	20%	27%	26%	24%	34%	22%
glcm	Correlation	3%	10%	11%	8%	6%	15%	20%	11%	8%	17%	14%	6%	21%	40%	17%	19%	17%	12%	38%	30%	16%	6%	24%	34%	32%	19%	20%	10%
glcm	DifferenceAverage	7%	13%	10%	18%	10%	16%	21%	17%	28%	16%	17%	12%	26%	19%	22%	19%	47%	15%	15%	17%	19%	17%	12%	17%	16%	16%	22%	14%
glcm	DifferenceEntropy	5%	7%	5%	10%	6%	8%	11%	11%	16%	9%	9%	7%	14%	10%	12%	11%	32%	10%	8%	9%	12%	9%	7%	8%	9%	8%	13%	8%
glcm	DifferenceVariance	19%	19%	15%	23%	17%	19%	26%	26%	47%	24%	21%	19%	31%	25%	24%	25%	36%	25%	21%	23%	30%	17%	17%	21%	21%	18%	27%	18%
glcm	Id	2%	5%	5%	8%	4%	7%	7%	7%	7%	5%	5%	4%	11%	7%	7%	8%	10%	6%	7%	6%	5%	5%	5%	7%	7%	5%	9%	5%
glcm	Idm	2%	5%	6%	10%	5%	9%	8%	9%	8%	7%	6%	5%	15%	10%	8%	11%	10%	8%	9%	8%	7%	5%	7%	10%	9%	6%	11%	6%
glcm	Idmn	0%	0%	1%	1%	1%	1%	1%	1%	1%	1%	0%	0%	1%	1%	0%	1%	0%	1%	1%	1%	0%	0%	1%	1%	1%	0%	1%	0%
glcm	Idn	1%	1%	1%	2%	1%	2%	2%	2%	2%	1%	1%	1%	3%	2%	2%	2%	2%	2%	2%	2%	1%	1%	1%	2%	2%	1%	2%	1%
glcm	Imc1	8%	20%	21%	21%	14%	33%	34%	37%	23%	31%	29%	14%	40%	79%	29%	54%	30%	35%	61%	43%	30%	16%	44%	68%	45%	31%	32%	21%
glcm	Imc2	2%	7%	8%	7%	5%	13%	16%	8%	6%	14%	11%	5%	13%	29%	14%	15%	24%	9%	27%	22%	13%	5%	17%	26%	20%	16%	16%	9%
glcm	InverseVariance	10%	9%	4%	5%	4%	3%	5%	6%	8%	5%	8%	6%	6%	5%	8%	4%	41%	6%	5%	4%	6%	11%	3%	4%	4%	5%	7%	3%
glcm	JointAverage	3%	8%	4%	9%	3%	10%	18%	6%	8%	9%	14%	7%	8%	11%	15%	6%	38%	8%	12%	11%	11%	14%	9%	6%	10%	12%	13%	10%
glcm	JointEnergy	21%	21%	18%	53%	16%	37%	37%	15%	16%	25%	17%	17%	48%	32%	38%	32%	70%	17%	32%	30%	15%	28%	27%	22%	32%	31%	62%	35%
glcm	JointEntropy	2%	6%	4%	9%	4%	7%	12%	4%	6%	7%	5%	5%	9%	8%	13%	7%	42%	4%	7%	9%	4%	10%	6%	5%	7%	9%	14%	8%
glcm	MCC	3%	8%	10%	8%	6%	15%	19%	11%	8%	16%	14%	6%	18%	35%	15%	18%	14%	12%	33%	26%	16%	6%	22%	30%	24%	18%	18%	10%
glcm	MaximumProbability	20%	22%	20%	44%	17%	39%	35%	16%	18%	24%	18%	20%	52%	30%	35%	32%	47%	18%	31%	29%	20%	26%	28%	23%	29%	30%	49%	31%
glcm	SumAverage	3%	8%	4%	9%	3%	10%	18%	6%	8%	9%	14%	7%	8%	11%	15%	6%	38%	8%	12%	11%	11%	14%	9%	6%	10%	12%	13%	10%
glcm	SumEntropy	2%	5%	3%	6%	3%	6%	10%	2%	2%	6%	4%	3%	4%	8%	10%	3%	39%	2%	7%	7%	5%	8%	5%	5%	6%	8%	11%	6%
glcm	SumSquares	10%	18%	14%	24%	15%	23%	39%	13%	16%	27%	15%	16%	22%	31%	37%	17%	59%	13%	36%	23%	20%	22%	16%	18%	18%	27%	34%	25%
gldm	DependenceEntropy	2%	3%	2%	3%	1%	3%	4%	2%	3%	4%	3%	3%	2%	6%	5%	2%	26%	2%	3%	3%	4%	4%	2%	4%	3%	4%	5%	3%
gldm	DependenceNonUniformity	6%	11%	7%	10%	6%	11%	11%	6%	7%	8%	8%	9%	13%	10%	11%	10%	68%	6%	9%	11%	7%	6%	7%	10%	9%	9%	11%	8%
gldm	DependenceNonUniformityNormalizec	6%	11%	7%	10%	6%	11%	11%	6%	7%	8%	8%	9%	13%	10%	11%	10%	68%	6%	9%	11%	7%	6%	7%	10%	9%	9%	11%	8%
gldm	DependenceVariance	14%	18%	16%	18%	11%	23%	18%	11%	11%	15%	14%	15%	24%	21%	18%	22%	19%	11%	22%	18%	12%	11%	16%	20%	17%	16%	22%	13%
gldm	GrayLevelNonUniformity	9%	11%	8%	21%	8%	16%	20%	5%	7%	14%	9%	10%	17%	17%	20%	11%	38%	6%	14%	15%	11%	15%	11%	11%	12%	17%	26%	18%
gldm	GrayLevelVariance	10%	18%	14%	24%	15%	23%	39%	13%	16%	27%	15%	16%	22%	31%	37%	17%	59%	13%	36%	23%	20%	22%	16%	18%	18%	27%	34%	25%
gldm	HighGrayLevelEmphasis	5%	14%	7%	15%	6%	16%	37%	10%	15%	17%	27%	13%	15%	19%	29%	12%	74%	13%	24%	20%	19%	27%	17%	10%	19%	24%	22%	18%
gldm	LargeDependenceEmphasis	9%	18%	15%	30%	12%	27%	23%	21%	21%	18%	18%	14%	40%	27%	25%	32%	34%	16%	27%	22%	19%	17%	21%	25%	26%	20%	34%	18%
gldm	LargeDependenceHighGrayLevelEmpha	15%	29%	19%	21%	14%	22%	34%	11%	21%	25%	29%	18%	29%	35%	39%	20%	49%	14%	62%	37%	22%	24%	24%	28%	43%	26%	35%	27%
gldm	LargeDependenceLowGrayLevelEmpha	29%	20%	25%	74%	22%	90%	56%	51%	38%	34%	38%	36%	50%	113%	52%	73%	94%	53%	22%	28%	41%	42%	37%	60%	56%	39%	101%	39%
gldm	LowGrayLevelEmphasis	18%	25%	16%	29%	18%	38%	37%	19%	20%	27%	29%	23%	22%	50%	35%	23%	61%	22%	24%	35%	27%	29%	25%	27%	29%	28%	44%	29%
gldm	SmallDependenceEmphasis	9%	19%	14%	21%	13%	20%	24%	19%	34%	21%	24%	16%	30%	23%	25%	22%	44%	17%	19%	23%	23%	22%	16%	23%	21%	21%	25%	17%
gldm	SmallDependenceHighGrayLevelEmpha	8%	17%	14%	25%	12%	26%	44%	20%	40%	28%	39%	21%	36%	27%	37%	25%	87%	21%	20%	23%	32%	36%	24%	27%	23%	31%	30%	23%
gldm	SmallDependenceLowGrayLevelEmpha	17%	36%	25%	28%	25%	28%	40%	25%	35%	33%	34%	28%	33%	36%	38%	29%	27%	21%	34%	46%	32%	28%	32%	29%	38%	34%	42%	29%
glrlm	GrayLevelNonUniformity	5%	10%	5%	5%	5%	6%	9%	7%	14%	10%	12%	8%	7%	12%	9%	6%	26%	6%	9%	6%	17%	9%	5%	9%	6%	8%	8%	9%
glrlm	GrayLevelNonUniformityNormalized	3%	9%	7%	16%	7%	12%	16%	5%	6%	12%	7%	8%	12%	14%	16%	8%	27%	6%	11%	11%	10%	11%	9%	8%	11%	12%	20%	14%
glrlm	GrayLevelVariance	9%	15%	12%	20%	13%	18%	30%	11%	13%	22%	13%	14%	17%	24%	29%	14%	31%	12%	27%	19%	18%	17%	14%	14%	16%	21%	29%	20%
glrlm	HighGrayLevelRunEmphasis	4%	13%	7%	14%	5%	16%	34%	8%	12%	17%	26%	12%	14%	18%	28%	11%	61%	11%	21%	19%	17%	23%	17%	10%	18%	23%	22%	17%
glrlm	LongRunEmphasis	9%	27%	12%	42%	9%	28%	30%	25%	31%	19%	29%	17%	56%	32%	38%	38%	220%	18%	30%	26%	27%	32%	25%	22%	37%	25%	70%	22%
glrlm	LongRunHighGrayLevelEmphasis	15%	38%	16%	21%	12%	19%	32%	11%	30%	26%	33%	19%	39%	39%	54%	22%	118%	11%	64%	41%	27%	28%	24%	25%	44%	27%	39%	30%
glrlm	LongRunLowGrayLevelEmphasis	28%	21%	18%	93%	17%	88%	64%	50%	44%	33%	44%	36%	68%	112%	61%	79%	256%	49%	20%	28%	45%	53%	39%	48%	89%	42%	165%	36%
glrlm	LowGrayLevelRunEmphasis	14%	23%	15%	28%	17%	37%	37%	17%	17%	27%	29%	21%	23%	41%	36%	23%	49%	20%	24%	33%	26%	29%	26%	25%	29%	28%	47%	25%
glrlm	RunEntropy	1%	4%	2%	2%	1%	3%	3%	4%	6%	4%	5%	3%	6%	6%	4%	4%	5%	3%	4%	3%	6%	3%	3%	5%	4%	3%	3%	3%
glrlm	RunLengthNonUniformity	5%	22%	10%	19%	9%	18%	24%	15%	31%	17%	24%	15%	27%	20%	28%	19%	65%	13%	16%	21%	23%	27%	13%	18%	17%	21%	25%	16%
glrlm	RunLengthNonUniformityNormalized	3%	12%	6%	12%	5%	11%	14%	10%	18%	10%	14%	9%	18%	13%	16%	12%	36%	8%	10%	13%	14%	15%	8%	11%	11%	12%	15%	10%
glrlm	RunPercentage	3%	10%	4%	9%	3%	8%	11%	7%	13%	7%	11%	7%	13%	9%	13%	9%	40%	6%	7%	9%	10%	12%	6%	8%	8%	9%	12%	7%
glrlm	RunVariance	16%	38%	24%	71%	17%	50%	44%	42%	39%	30%	39%	25%	92%	51%	55%	69%	179%	30%	55%	41%	37%	42%	49%	39%	66%	38%	128%	35%
glrlm	ShortRunEmphasis	1%	7%	3%	7%	3%	6%	8%	6%	11%	6%	8%	5%	10%	7%	9%	7%	24%	4%	6%	7%	8%	9%	4%	6%	6%	7%	9%	6%
glrlm	ShortRunHighGrayLevelEmphasis	4%	11%	7%	17%	6%	18%	36%	11%	18%	18%	29%	13%	21%	18%	29%	15%	67%	13%	15%	16%	20%	26%	18%	13%	16%	25%	22%	17%
glrlm	ShortRunLowGrayLevelEmphasis	13%	25%	16%	23%	18%	29%	34%	15%	19%	27%	28%	21%	22%	32%	34%	19%	38%	17%	25%	36%	25%	25%	26%	21%	29%	27%	39%	24%
glszm	GrayLevelNonUniformity	7%	20%	13%	21%	11%	20%	26%	21%	48%	24%	28%	16%	33%	22%	27%	23%	34%	19%	18%	22%	35%	23%	15%	22%	20%	22%	22%	19%
glszm	GrayLevelNonUniformityNormalized	4%	7%	4%	8%	4%	7%	12%	4%	8%	7%	7%	6%	7%	10%	14%	5%	46%	5%	8%	11%	7%	12%	7%	5%	9%	13%	25%	7%
glszm	GrayLevelVariance	6%	12%	7%	10%	8%	9%	11%	8%	9%	11%	10%	10%	11%	11%	13%	8%	24%	10%	9%	11%	13%	11%	9%	8%	10%	10%	15%	10%
glszm	HighGrayLevelZoneEmphasis	3%	11%	7%	11%	5%	12%	23%	5%	8%	15%	20%	10%	13%	16%	23%	10%	36%	8%	14%	15%	13%	16%	15%	10%	15%	18%	20%	14%
glszm	LargeAreaEmphasis	21%	65%	65%	344%	46%	237%	116%	206%	62%	112%	58%	56%	195%	176%	91%	254%	171%	231%	235%	110%	63%	81%	237%	106%	353%	102%	206%	173%
glszm	LargeAreaHighGrayLevelEmphasis	27%	73%	73%	238%	45%	138%	94%	158%	65%	123%	81%	61%	173%	233%	107%	181%	87%	136%	313%	121%	60%	76%	193%	110%	279%	99%	135%	187%
glszm	LargeAreaLowGrayLevelEmphasis	45%	61%	50%	487%	31%	424%	166%	255%	66%	110%	73%	49%	210%	142%	114%	378%	215%	310%	157%	100%	74%	100%	302%	102%	717%	116%	349%	178%
glszm	LowGrayLevelZoneEmphasis	9%	22%	15%	31%	15%	34%	39%	17%	20%	30%	30%	19%	32%	28%	39%	27%	39%	24%	23%	29%	28%	28%	28%	23%	32%	31%	51%	23%
glszm	SizeZoneNonUniformity	21%	35%	26%	34%	25%	34%	41%	33%	69%	44%	44%	32%	46%	39%	37%	35%	60%	29%	31%	39%	41%	41%	27%	40%	36%	36%	37%	32%
glszm	SizeZoneNonUniformityNormalized	14%	17%	12%	14%	13%	14%	22%	15%	23%	21%	19%	16%	24%	16%	18%	15%	38%	12%	15%	17%	18%	21%	13%	20%	17%	20%	16%	17%
glszm	SmallAreaEmphasis	8%	11%	8%	9%	8%	9%	13%	10%	14%	12%	12%	10%	15%	9%	11%	10%	20%	8%	9%	10%	11%	16%	8%	13%	10%	12%	10%	11%
glszm	SmallAreaHighGrayLevelEmphasis	9%	17%	10%	15%	9%	15%	20%	11%	17%	20%	26%	14%	23%	19%	22%	15%	37%	12%	14%	19%	17%	24%	18%	20%	15%	18%	22%	21%
glszm	SmallAreaLowGrayLevelEmphasis	18%	28%	21%	31%	21%	32%	46%	23%	35%	30%	33%	27%	29%	25%	40%	25%	41%	29%	28%	29%	33%	40%	30%	23%	37%	36%	47%	27%
glszm	ZoneEntropy	3%	3%	2%	2%	2%	3%	6%	2%	3%	4%	3%	3%	5%	4%	6%	2%	17%	2%	3%	5%	4%	4%	3%	5%	4%	5%	6%	3%
glszm	ZonePercentage	9%	22%	15%	24%	13%	23%	33%	22%	51%	25%	30%	19%	33%	27%	33%	25%	55%	20%	20%	27%	32%	27%	18%	24%	23%	26%	31%	21%
glszm	ZoneVariance	27%	70%	81%	368%	58%	264%	120%	232%	62%	124%	59%	61%	201%	186%	92%	270%	164%	257%	251%	114%	64%	83%	262%	119%	368%	107%	210%	194%
ngtdm	Busyness	11%	16%	12%	14%	12%	15%	21%	11%	16%	15%	15%	15%	24%	20%	17%	14%	46%	14%	20%	21%	17%	16%	14%	17%	19%	16%	24%	14%
ngtdm	Coarseness	15%	13%	8%	8%	8%	14%	15%	17%	19%	11%	12%	7%	14%	21%	10%	13%	141%	13%	11%	10%	18%	10%	9%	21%	11%	10%	9%	12%
ngtdm	Complexity	13%	12%	10%	13%	10%	15%	15%	17%	22%	12%	14%	10%	20%	18%	13%	17%	43%	16%	12%	15%	20%	11%	11%	18%	14%	12%	15%	12%
ngtdm	Contrast	19%	24%	22%	33%	25%	31%	52%	27%	31%	36%	20%	24%	40%	40%	49%	32%	70%	26%	39%	36%	16%	31%	25%	28%	29%	36%	47%	31%
ngtdm	Strength	8%	18%	12%	16%	10%	16%	19%	12%	18%	12%	17%	9%	27%	26%	18%	18%	160%	9%	29%	21%	19%	20%	15%	23%	21%	15%	20%	11%

20%
15%
10%
5%
0%

(B) Heatmap of reproducibility of radiomics features according to materials by QCD

Family	Feature	ROI_1	ROI_2	ROI_3	ROI_4	ROI_5	ROI_6	ROI_7	ROI_8	ROI_9	ROI_10	ROI_11	ROI_12	ROI_13	ROI_14	ROI_15	ROI_16	ROI_17	ROI_18	ROI_19	ROI_20	ROI_21	ROI_22	ROI_23	ROI_24	ROI_25	ROI_26	ROI_27	ROI_28
firstorder	10Percentile	4%	1%	5%	8%	8%	9%	2%	8%	0%	2%	2%	0%	2%	7%	10%	88%	29%	8%	0%	12%	0%	1%	0%	6%	57%	1%	2%	3%
firstorder	90Percentile	2%	1%	31%	23%	22%	11%	2%	8%	0%	2%	6%	1%	2%	17%	50%	5%	10%	7%	0%	20%	0%	2%	0%	26%	19%	1%	3%	3%
firstorder	Energy	8%	1%	10%	14%	14%	18%	4%	14%	0%	3%	5%	1%	1%	8%	23%	8%	24%	12%	0%	17%	0%	3%	0%	7%	35%	1%	1%	7%
firstorder	Entropy	1%	4%	2%	2%	2%	3%	8%	1%	2%	3%	3%	3%	5%	5%	10%	3%	27%	2%	3%	6%	2%	6%	3%	4%	4%	7%	8%	3%
firstorder	InterquartileRange	7%	12%	8%	9%	11%	10%	13%	8%	3%	9%	9%	7%	19%	13%	13%	13%	10%	7%	36%	24%	9%	8%	19%	8%	27%	11%	20%	13%
firstorder	Kurtosis	5%	10%	4%	5%	5%	7%	17%	4%	5%	6%	6%	4%	14%	11%	19%	6%	79%	5%	5%	18%	4%	25%	4%	6%	6%	15%	16%	7%
firstorder	Maximum	5%	3%	11%	13%	8%	9%	4%	6%	2%	6%	16%	4%	7%	364%	40%	5%	42%	6%	1%	243%	1%	8%	1%	55%	20%	3%	16%	15%
firstorder	MeanAbsoluteDeviation	5%	10%	7%	8%	10%	10%	13%	7%	2%	10%	8%	4%	13%	11%	16%	11%	11%	6%	33%	27%	8%	8%	18%	9%	26%	12%	20%	11%
firstorder	Mean	9%	0%	4%	11%	4%	8%	2%	11%	0%	2%	3%	0%	0%	2%	20%	3%	16%	16%	0%	8%	0%	2%	0%	2%	7%	1%	1%	2%
firstorder	Median	20%	1%	6%	16%	8%	14%	2%	25%	0%	2%	2%	0%	1%	2%	23%	3%	19%	45%	0%	7%	0%	2%	0%	3%	6%	1%	1%	3%
firstorder	Minimum	1%	5%	7%	6%	5%	9%	3%	5%	1%	5%	3%	1%	3%	10%	23%	18%	17%	5%	1%	19%	1%	2%	1%	6%	35%	2%	10%	3%
firstorder	Range	2%	14%	7%	7%	5%	9%	22%	5%	7%	15%	10%	7%	6%	25%	27%	9%	37%	5%	31%	33%	17%	11%	20%	14%	25%	17%	29%	8%
firstorder	RobustMeanAbsoluteDeviation	6%	11%	8%	9%	10%	10%	13%	7%	2%	10%	9%	6%	17%	13%	14%	12%	10%	7%	32%	25%	7%	8%	17%	8%	24%	11%	19%	12%
firstorder	RootMeanSquared	4%	0%	5%	7%	7%	9%	2%	7%	0%	2%	3%	0%	1%	4%	12%	4%	12%	6%	0%	9%	0%	2%	0%	4%	18%	1%	1%	4%
firstorder	Skewness	16%	19%	16%	14%	10%	10%	120%	7%	29%	79%	33%	46%	45%	34%	256%	10%	61%	8%	122%	45%	21%	34%	55%	22%	160%	67%	41%	43%
firstorder	TotalEnergy	11%	1%	11%	15%	15%	18%	4%	14%	0%	3%	5%	1%	1%	8%	23%	11%	24%	14%	0%	17%	0%	4%	0%	7%	39%	2%	1%	7%
firstorder	Uniformity	6%	7%	4%	5%	5%	6%	14%	3%	4%	6%	5%	6%	12%	9%	16%	6%	22%	4%	6%	11%	4%	9%	5%	7%	7%	12%	13%	7%
firstorder	Variance	8%	19%	14%	15%	18%	18%	27%	13%	5%	22%	17%	6%	21%	22%	32%	20%	32%	12%	59%	53%	18%	17%	36%	19%	48%	24%	39%	19%
glcm	Autocorrelation	4%	10%	4%	6%	4%	5%	24%	4%	8%	9%	10%	8%	9%	13%	22%	5%	54%	4%	11%	14%	8%	16%	11%	7%	11%	19%	12%	11%
glcm	ClusterProminence	10%	24%	12%	16%	18%	18%	43%	14%	20%	26%	25%	20%	19%	26%	47%	14%	30%	14%	21%	31%	28%	26%	19%	18%	23%	37%	38%	25%
glcm	ClusterShade	17%	29%	19%	22%	18%	17%	235%	15%	31%	72%	42%	175%	82%	29%	110%	15%	26%	15%	97%	37%	36%	35%	79%	21%	193%	63%	49%	40%
glcm	ClusterTendency	6%	13%	7%	8%	10%	10%	27%	7%	11%	14%	14%	11%	14%	15%	31%	8%	41%	7%	11%	19%	15%	16%	11%	10%	13%	23%	23%	13%
glcm	Contrast	9%	12%	10%	14%	12%	14%	19%	14%	17%	19%	20%	13%	28%	23%	22%	14%	36%	12%	13%	19%	20%	16%	12%	16%	16%	16%	23%	14%
glcm	Correlation	1%	5%	6%	5%	4%	7%	12%	3%	5%	10%	12%	3%	7%	21%	11%	8%	10%	4%	17%	18%	6%	5%	11%	16%	18%	12%	12%	5%
glcm	DifferenceAverage	4%	8%	6%	9%	7%	8%	12%	7%	13%	12%	12%	7%	16%	13%	16%	8%	34%	7%	7%	12%	13%	11%	7%	11%	10%	11%	14%	8%
glcm	DifferenceEntropy	2%	4%	3%	4%	3%	4%	6%	4%	6%	6%	7%	4%	8%	7%	8%	4%	19%	4%	4%	6%	8%	6%	4%	5%	5%	5%	7%	5%
glcm	DifferenceVariance	11%	12%	8%	11%	10%	11%	15%	12%	11%	16%	16%	14%	19%	18%	16%	12%	23%	10%	11%	15%	15%	12%	10%	11%	14%	13%	15%	11%
glcm	Id	1%	3%	3%	3%	3%	4%	4%	3%	4%	4%	3%	2%	6%	4%	5%	4%	7%	3%	3%	4%	4%	3%	3%	5%	4%	4%	5%	3%
glcm	Idm	1%	3%	4%	5%	4%	5%	5%	4%	5%	5%	4%	3%	8%	6%	6%	5%	8%	4%	4%	5%	5%	3%	4%	6%	5%	4%	7%	4%
glcm	Idmn	0%	0%	0%	0%	0%	0%	0%	0%	0%	0%	0%	0%	1%	1%	0%	0%	0%	0%	0%	0%	0%	0%	0%	0%	0%	0%	0%	0%
glcm	Idn	0%	1%	1%	1%	1%	1%	1%	1%	1%	1%	1%	1%	2%	1%	1%	1%	2%	1%	1%	1%	1%	1%	1%	1%	1%	1%	1%	1%
glcm	Imc1	4%	11%	13%	13%	9%	17%	27%	8%	17%	22%	24%	9%	7%	37%	22%	14%	16%	10%	25%	35%	18%	9%	17%	27%	27%	21%	22%	13%
glcm	Imc2	1%	4%	5%	4%	3%	6%	10%	3%	3%	9%	9%	3%	2%	18%	9%	6%	15%	4%	13%	15%	4%	4%	8%	12%	12%	10%	9%	4%
glcm	InverseVariance	6%	7%	2%	2%	3%	2%	3%	4%	7%	3%	6%	5%	4%	3%	6%	2%	27%	4%	2%	3%	4%	7%	2%	2%	2%	4%	3%	2%
glcm	JointAverage	2%	6%	2%	3%	2%	3%	12%	2%	4%	5%	6%	5%	5%	7%	11%	3%	30%	2%	6%	8%	4%	9%	6%	4%	6%	9%	6%	6%
glcm	JointEnergy	15%	14%	9%	13%	10%	14%	25%	8%	10%	15%	10%	10%	22%	16%	29%	13%	41%	8%	11%	20%	10%	18%	10%	12%	13%	22%	26%	14%
glcm	JointEntropy	1%	4%	2%	3%	2%	4%	8%	2%	3%	4%	3%	3%	5%	5%	10%	3%	28%	2%	3%	6%	3%	6%	3%	3%	4%	7%	8%	4%
glcm	MCC	1%	4%	5%	5%	4%	7%	11%	3%	4%	10%	11%	3%	3%	20%	10%	7%	9%	4%	16%	18%	5%	4%	10%	14%	15%	11%	11%	5%
glcm	MaximumProbability	12%	15%	10%	17%	12%	17%	23%	12%	13%	14%	11%	13%	25%	16%	27%	16%	32%	12%	13%	20%	14%	17%	12%	14%	15%	21%	25%	15%
glcm	SumAverage	2%	6%	2%	3%	2%	3%	12%	2%	4%	5%	6%	5%	5%	7%	11%	3%	30%	2%	6%	8%	4%	9%	6%	4%	6%	9%	6%	6%
glcm	SumEntropy	1%	3%	1%	2%	2%	2%	7%	1%	2%	3%	3%	2%	3%	4%	8%	2%	25%	1%	3%	5%	2%	4%	3%	2%	3%	6%	6%	3%
glcm	SumSquares	7%	13%	7%	9%	10%	11%	24%	8%	10%	12%	11%	11%	20%	15%	27%	10%	41%	8%	11%	17%	13%	15%	10%	11%	12%	20%	23%	12%
gldm	DependenceEntropy	1%	2%	1%	1%	1%	1%	3%	1%	2%	2%	2%	2%	1%	2%	4%	1%	12%	1%	2%	2%	2%	2%	1%	1%	2%	2%	2%	1%
gldm	DependenceNonUniformity	4%	8%	5%	6%	3%	7%	8%	3%	5%	6%	5%	6%	7%	6%	8%	6%	12%	4%	5%	7%	5%	4%	4%	7%	6%	7%	8%	6%
gldm	DependenceNonUniformityNormalizec	4%	8%	5%	6%	3%	7%	8%	3%	5%	6%	5%	6%	7%	6%	8%	6%	12%	4%	5%	7%	5%	4%	4%	7%	6%	7%	8%	6%
gldm	DependenceVariance	9%	14%	10%	11%	8%	15%	13%	7%	8%	11%	10%	11%	12%	10%	15%	12%	9%	8%	10%	14%	9%	7%	8%	14%	10%	12%	15%	10%
gldm	GrayLevelNonUniformity	6%	7%	4%	5%	5%	6%	14%	3%	4%	6%	5%	6%	12%	9%	16%	6%	22%	4%	6%	11%	4%	9%	5%	7%	7%	12%	13%	7%
gldm	GrayLevelVariance	7%	12%	7%	9%	10%	11%	23%	8%	10%	12%	11%	11%	20%	15%	28%	10%	41%	8%	11%	17%	13%	15%	10%	11%	12%	20%	24%	12%
gldm	HighGrayLevelEmphasis	4%	10%	4%	5%	4%	5%	24%	4%	7%	9%	11%	8%	9%	12%	22%	5%	53%	4%	10%	14%	7%	16%	11%	7%	10%	19%	11%	11%
gldm	LargeDependenceEmphasis	6%	13%	10%	11%	7%	13%	15%	6%	16%	13%	13%	10%	16%	13%	20%	11%	25%	6%	9%	16%	15%	11%	9%	17%	12%	14%	19%	11%
gldm	LargeDependenceHighGrayLevelEmpha	10%	23%	10%	14%	10%	14%	22%	8%	15%	15%	16%	12%	18%	19%	18%	10%	41%	8%	16%	25%	17%	15%	14%	17%	17%	16%	27%	14%
gldm	LargeDependenceLowGrayLevelEmpha	20%	10%	12%	14%	16%	17%	32%	15%	26%	23%	27%	25%	19%	17%	30%	13%	70%	13%	12%	17%	26%	31%	20%	14%	15%	28%	14%	24%
gldm	LowGrayLevelEmphasis	11%	16%	11%	13%	13%	12%	25%	10%	14%	19%	19%	17%	15%	20%	25%	10%	54%	9%	14%	21%	17%	22%	17%	13%	15%	20%	19%	18%
gldm	SmallDependenceEmphasis	6%	11%	9%	11%	9%	13%	15%	9%	19%	16%	16%	10%	20%	15%	19%	11%	34%	8%	8%	17%	17%	14%	9%	17%	12%	14%	17%	12%
gldm	SmallDependenceHighGrayLevelEmpha	5%	10%	9%	11%	9%	17%	26%	10%	20%	20%	24%	12%	22%	14%	24%	13%	62%	10%	12%	15%	19%	23%	16%	18%	14%	23%	15%	17%
gldm	SmallDependenceLowGrayLevelEmpha	12%	22%	15%	18%	18%	18%	25%	17%	24%	24%	22%	19%	23%	22%	27%	20%	18%	15%	20%	31%	22%	18%	23%	19%	25%	24%	31%	20%
glrlm	GrayLevelNonUniformity	3%	7%	2%	3%	3%	2%	6%	2%	13%	7%	11%	6%	4%	4%	6%	3%	12%	3%	4%	4%	10%	5%	3%	3%	4%	5%	5%	5%
glrlm	GrayLevelNonUniformityNormalized	2%	5%	4%	4%	5%	6%	11%	3%	4%	6%	5%	5%	9%	8%	12%	4%	19%	4%	5%	8%	4%	8%	5%	6%	5%	9%	11%	6%
glrlm	GrayLevelVariance	6%	10%	7%	8%	9%	10%	19%	7%	8%	11%	9%	9%	14%	14%	22%	8%	21%	8%	9%	14%	12%	11%	9%	10%	10%	15%	20%	11%
glrlm	HighGrayLevelRunEmphasis	3%	9%	4%	5%	3%	5%	23%	3%	6%	9%	10%	8%	9%	12%	21%	5%	43%	3%	10%	14%	7%	13%	10%	7%	10%	17%	11%	11%
glrlm	LongRunEmphasis	7%	18%	7%	9%	6%	11%	18%	6%	24%	13%	21%	13%	14%	12%	26%	10%	60%	6%	8%	16%	20%	19%	7%	14%	11%	17%	19%	11%
glrlm	LongRunHighGrayLevelEmphasis	11%	28%	9%	12%	8%	11%	21%	7%	23%	15%	23%	14%	15%	17%	21%	9%	26%	7%	14%	25%	21%	16%	13%	14%	15%	17%	26%	14%
glrlm	LongRunLowGrayLevelEmphasis	18%	11%	11%	12%	12%	14%	34%	10%	32%	21%	29%	24%	17%	16%	33%	10%	82%	10%	11%	17%	29%	34%	18%	12%	15%	29%	14%	22%
glrlm	LowGrayLevelRunEmphasis	9%	15%	10%	12%	12%	12%	26%	9%	12%	18%	19%	15%	16%	19%	25%	10%	45%	9%	14%	21%	17%	19%	17%	12%	15%	20%	18%	17%
glrlm	RunEntropy	1%	2%	1%	1%	1%	2%	3%	1%	5%	3%	5%	2%	2%	2%	3%	1%	3%	1%	1%	3%	4%	2%	1%	2%	2%	2%	2%	2%
glrlm	RunLengthNonUniformity	4%	15%	7%	9%	6%	10%	16%	6%	21%	13%	17%	10%	14%	12%	22%	8%	53%	5%	6%	15%	18%	18%	7%	13%	10%	15%	17%	11%
glrlm	RunLengthNonUniformityNormalized	2%	8%	4%	5%	3%	6%	9%	3%	13%	8%	10%	6%	9%	7%	13%	5%	28%	3%	4%	9%	11%	11%	4%	8%	6%	9%	10%	6%
glrlm	RunPercentage	2%	7%	3%	4%	2%	4%	7%	2%	9%	5%	7%	5%	5%	5%	10%	3%	28%	2%	3%	6%	8%	8%	3%	5%	4%	6%	7%	4%
glrlm	RunVariance	11%	26%	15%	17%	11%	19%	27%	10%	30%	20%	28%	18%	24%	21%	38%	19%	66%	11%	15%	25%	26%	25%	13%	22%	18%	25%	29%	18%
glrlm	ShortRunEmphasis	1%	5%	2%	3%	2%	3%	5%	2%	8%	4%	6%	3%	4%	3%	7%	2%	18%	2%	2%	5%	6%	6%	2%	4%	3%	5%	5%	3%
glrlm	ShortRunHighGrayLevelEmphasis	3%	7%	5%	6%	4%	8%	25%	4%	8%	10%	13%	9%	10%	10%	23%	6%	42%	4%	9%	12%	9%	16%	12%	7%	10%	20%	10%	11%
glrlm	ShortRunLowGrayLevelEmphasis	8%	17%	11%	13%	13%	13%	24%	10%	13%	18%	20%	15%	15%	21%	23%	10%	29%	9%	15%	23%	16%	15%	17%	13%	16%	20%	23%	16%
glszm	GrayLevelNonUniformity	5%	13%	8%	10%	7%	13%	16%	7%	23%	17%	21%	10%	20%	16%	19%	10%	19%	7%	7%	18%	23%	13%	8%	16%	12%	15%	15%	13%
glszm	GrayLevelNonUniformityNormalized	2%	5%	3%	4%	3%	4%	7%	3%	4%	5%	4%	4%	4%	6%	9%	3%	29%	3%	5%	8%	5%	7%	4%	3%	5%	7%	9%	3%
glszm	GrayLevelVariance	4%	8%	5%	7%	5%	6%	8%	5%	6%	8%	7%	7%	8%	8%	10%	5%	15%	6%	6%	7%	8%	7%	5%	6%	7%	7%	9%	6%
glszm	HighGrayLevelZoneEmphasis	2%	7%	4%	5%	4%	6%	18%	3%	5%	8%	10%	7%	8%	10%	16%	5%	25%	4%	9%	11%	8%	10%	10%	6%	9%	12%	9%	8%
glszm	LargeAreaEmphasis	13%	41%	29%	37%	25%	40%	69%	24%	41%	43%	45%	29%	53%	63%	72%	50%	70%	28%	45%	63%	44%	52%	37%	47%	44%	61%	78%	41%
glszm	LargeAreaHighGrayLevelEmphasis	17%	44%	28%	40%	24%	41%	56%	22%	43%	43%	32%	35%	49%	61%	64%	51%	39%	25%	41%	68%	39%	46%	37%	47%	46%	58%	81%	42%
glszm	LargeAreaLowGrayLevelEmphasis	24%	35%	26%	26%	20%	40%	77%	22%	43%	42%	53%	29%	40%	59%	74%	44%	92%	24%	43%	55%	53%	59%	43%	48%	41%	63%	72%	39%
glszm	LowGrayLevelZoneEmphasis	6%	14%	9%	10%	11%	12%	27%	8%	15%	18%	19%	14%	20%	18%	26%	11%	26%	10%	14%	21%	18%	19%	19%	12%	17%	23%	19%	16%
glszm	SizeZoneNonUniformity	15%	23%	17%	20%	18%	24%	29%	20%	37%	28%	33%	22%	38%	27%	27%	19%	43%	17%	14%	29%	28%	27%	16%	29%	22%	26%	25%	23%
glszm	SizeZoneNonUniformityNormalized	10%	11%	8%	9%	9%	10%	13%	10%	15%	11%	13%	11%	18%	10%	10%	9%	14%	8%	8%	11%	10%	13%	7%	12%	9%	11%	9%	12%
glszm	SmallAreaEmphasis	6%	8%	5%	5%	5%	7%	8%	6%	9%	7%	8%	6%	10%	6%	6%	6%	9%	5%	5%	7%	6%	10%	4%	7%	6%	7%	6%	7%
glszm	SmallAreaHighGrayLevelEmphasis	6%	10%	7%	9%	6%	10%	14%	6%	10%	13%	17%	10%	14%	11%	15%	9%	23%	8%	9%	13%	12%	17%	12%	10%	10%	13%	12%	13%
glszm	SmallAreaLowGrayLevelEmphasis	12%	18%	15%	15%	15%	15%	31%	14%	23%	19%	24%	18%	19%	16%	29%	16%	27%	15%	20%	22%	23%	26%	20%	14%	22%	24%	22%	17%
glszm	ZoneEntropy	2%	2%	2%	1%	2%	2%	3%	2%	2%	2%	2%	2%	3%	3%	4%	2%	9%	2%	2%	3%	2%	3%	1%	3%	2%	3%	3%	2%
glszm	ZonePercentage	6%	13%	10%	13%	9%	13%	19%	9%	24%	19%	21%	12%	20%	19%	23%	11%	43%	8%	10%	20%	23%	18%	11%	17%	14%	19%	22%	14%
glszm	ZoneVariance	17%	43%	33%	44%	30%	47%	72%	30%	41%	45%	45%	31%	57%	66%	73%	57%	70%	33%	50%	65%	44%	54%	41%	52%	48%	64%	81%	46%
ngtdm	Busyness	6%	11%	7%	10%	8%	9%	13%	7%	12%	11%	9%	10%	19%	11%	12%	8%	27%	7%	9%	12%	12%	10%	9%	9%	11%	10%	13%	9%
ngtdm	Coarseness	9%	7%	4%	4%	4%	5%	7%	7%	17%	8%	11%	5%	8%	7%	8%	4%	10%	7%	4%	6%	11%	6%	4%	5%	6%	7%	6%	7%
ngtdm	Complexity	7%	6%	5%	6%	6%	12%	11%	9%	15%	8%	11%	6%	13%	9%	9%	7%	29%	10%	5%	10%	12%	6%	5%	8%	9%	8%	7%	7%
ngtdm	Contrast	12%	15%	13%	17%	16%	19%	30%	18%	12%	20%	12%	17%	35%	24%	38%	18%	44%	15%	17%	22%	10%	21%	15%	16%	18%	27%	31%	19%
ngtdm	Strength	4%	13%	6%	8%	6%	9%	11%	6%	16%	8%	12%	6%	10%	12%	9%	7%	33%	6%	6%	15%	13%	14%	6%	11%	10%	9%	12%	6%

20% 15% 10% 5% 0%
